# Downstream Antisense Transcription Predicts Genomic Features That Define the Specific Chromatin Environment at Mammalian Promoters

**DOI:** 10.1371/journal.pgen.1006224

**Published:** 2016-08-03

**Authors:** Christopher A. Lavender, Kimberly R. Cannady, Jackson A. Hoffman, Kevin W. Trotter, Daniel A. Gilchrist, Brian D. Bennett, Adam B. Burkholder, Craig J. Burd, David C. Fargo, Trevor K. Archer

**Affiliations:** 1 Epigenetics and Stem Cell Biology Laboratory, National Institute of Environmental Health Sciences, National Institutes of Health, Research Triangle Park, North Carolina, United States of America; 2 Integrative Bioinformatics, National Institute of Environmental Health Sciences, National Institutes of Health, Research Triangle Park, North Carolina, United States of America; 3 Department of Molecular Genetics, The Ohio State University, Columbus, Ohio, United States of America; 4 Wexner Medical Center, The Ohio State University, Columbus, Ohio, United States of America; Netherlands Cancer Institute, NETHERLANDS

## Abstract

Antisense transcription is a prevalent feature at mammalian promoters. Previous studies have primarily focused on antisense transcription initiating upstream of genes. Here, we characterize promoter-proximal antisense transcription downstream of gene transcription starts sites in human breast cancer cells, investigating the genomic context of downstream antisense transcription. We find extensive correlations between antisense transcription and features associated with the chromatin environment at gene promoters. Antisense transcription downstream of promoters is widespread, with antisense transcription initiation observed within 2 kb of 28% of gene transcription start sites. Antisense transcription initiates between nucleosomes regularly positioned downstream of these promoters. The nucleosomes between gene and downstream antisense transcription start sites carry histone modifications associated with active promoters, such as H3K4me3 and H3K27ac. This region is bound by chromatin remodeling and histone modifying complexes including SWI/SNF subunits and HDACs, suggesting that antisense transcription or resulting RNA transcripts contribute to the creation and maintenance of a promoter-associated chromatin environment. Downstream antisense transcription overlays additional regulatory features, such as transcription factor binding, DNA accessibility, and the downstream edge of promoter-associated CpG islands. These features suggest an important role for antisense transcription in the regulation of gene expression and the maintenance of a promoter-associated chromatin environment.

## Author Summary

Gene transcription is regulated by the coordinated interaction of genetic, epigenetic and trans-acting factors. The chromatin environment at gene promoters, including positioned nucleosomes that may display functional histone modifications, is a key regulator of gene expression, contributing to transcriptional activation and repression. In addition to sense-strand transcription of gene sequences, antisense transcription is prevalent at gene promoters. Often resulting in a short-lived non-coding RNA transcript, the function of antisense transcription is poorly understood. Using next-generation sequencing techniques, we characterized transcription in human breast cancer cells and found extensive correlations between antisense transcription and the chromatin environment at promoters. We found that downstream antisense transcription initiates from between regularly positioned nucleosomes and that those nucleosomes between sense and downstream antisense transcription start sites display histone modifications associated with active gene promoters. Chromatin remodelers and other protein complexes responsible for creation and maintenance of the promoter chromatin environment associate with this same region, suggesting an important role of antisense transcription in the regulation of gene expression.

## Introduction

The promoter region is intimately tied to the transcription of genes, providing the initial site of transcriptional machinery binding and assembly. Comprised of DNA elements in defined spatial arrangements [1], promoters also display key genomic features that facilitate gene regulation. Active promoters present a nucleosome-deprived region that allows for the association of RNA polymerase II (Pol II) and transcription factors [2]. Active promoters also possess distinct histone marks associated with gene expression [3].

Divergent transcription has emerged as a common feature of mammalian promoters [4–7]. In divergent transcription, an additional transcription event initiates upstream and antisense of a nearby gene promoter. Though divergent transcription at promoters may result in two different protein-coding transcripts in opposite orientations, often a short-lived non-coding RNA is transcribed anti-sense of a gene [8–10]. In divergent transcription at mammalian promoters, antisense transcription initiates at the upstream antisense transcription start site (uaTSS). The position of the uaTSS intersects with distinguishing features associated with promoters, with uaTSSs falling at the border of nucleosome-depleted regions where transcription factor binding may be enriched [11]. Additionally, uaTSSs tend to broadly coincide with the upstream edge of promoter-associated CpG islands [11].

In addition to divergent transcription, convergent transcription has been observed at genes in a variety of systems, ranging from fly and yeast to mammals [12–14]. Convergent transcription is possible in a variety of different gene structures, including non-overlapping genes on opposite strands and genes with internal promoters [13]. Promoter-proximal convergent transcription is common; analysis identified convergent transcription at roughly one quarter of queried genes [15]. Here, we describe genetic and epigenetic features at downstream antisense transcription start sites (daTSSs) associated with promoter-proximal convergent transcription in human T47D/A1-2 cells. We find that daTSSs coincide with the downstream edge of promoter-associated genomic features, such as promoter-associated histone marks. Though convergent transcription has been suggested as a repressive feature [15], we find that genes with observable daTSSs do not display lower gene expression in T47D/A1-2 cells. Despite this, coincidence of daTSSs with a variety of transregulatory factors, such as transcription factors and chromatin remodelers, suggests an intimate connection between antisense transcription and gene regulation.

## Results

### Antisense transcription is wide-spread across mammalian genes

To characterize transcription initiation in human T47D/A1-2 cells, Start-seq was performed on nascent RNA transcripts [16]. In a Start-seq experiment, RNA is isolated from the nucleus and selected for short size, allowing characterization of nascent RNA transcripts that may be subsequently degraded and that otherwise could not be analyzed in a traditional RNA-seq experiment. Cap-sensitive degradation ensures that the 5’ end of each read in a Start-seq experiment corresponds to a TSS with single nucleotide resolution. Gene TSSs and uaTSSs were identified using previously described methods [11]. daTSSs were called in a method analogous to that used for uaTSS identification. In brief, for each gene TSS called, a search window from the gene TSS to 2 kb downstream was defined. For each search window with reads exceeding an FDR-defined significance threshold (5 reads, observing aligned library depth), a daTSS was called at the position with greatest read density on the opposite strand relative to the gene TSS. Stringent filtering was used to ensure that an identified daTSS could not be a miscalled TSS of another gene or a uaTSS of an alternative start site.

**Fig 1:**
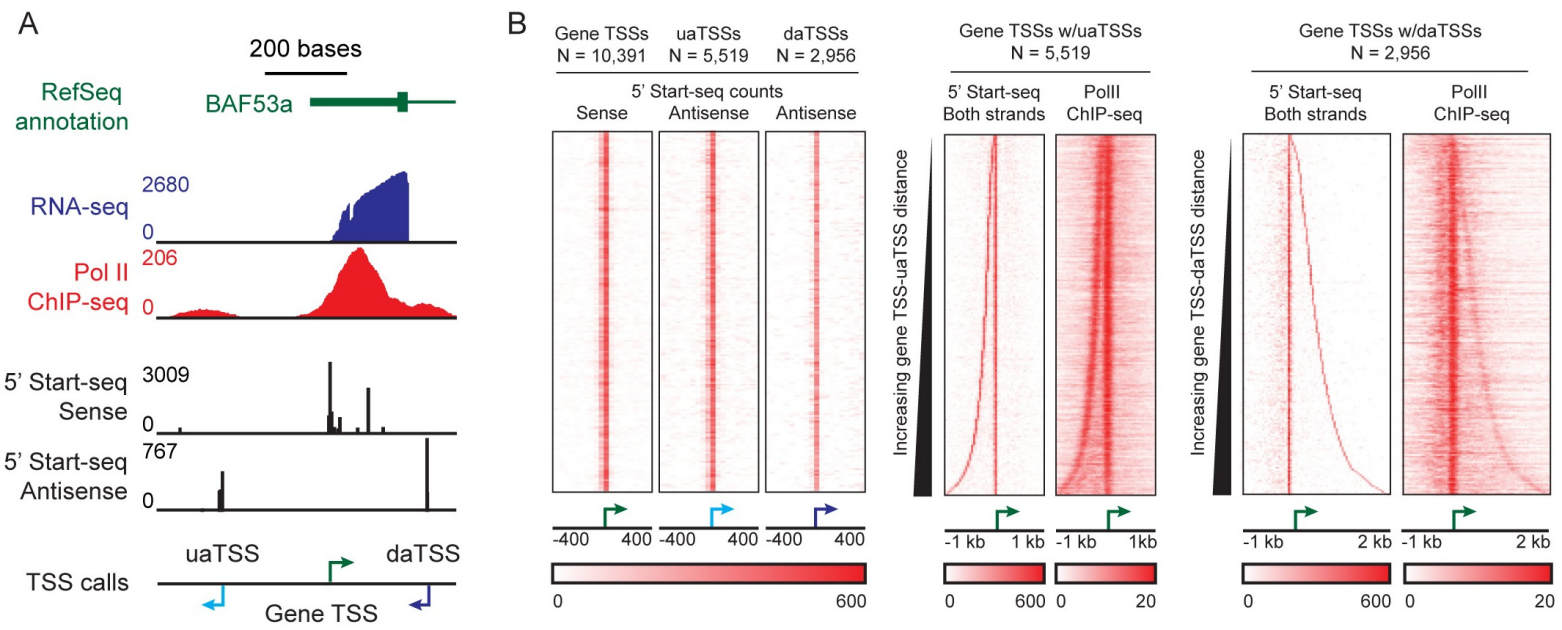
Antisense transcription is wide-spread across genes in human T47D/A1-2 cells. (A) Observed transcription at gene BAF53a. Genomic context is given by RefSeq annotation (green), RNA-seq coverage (blue), Pol II ChIP-seq coverage (red; from HMEC cells), and counts for the 5’ ends of Start-seq reads in both sense and anti-sense orientations (black). Observed transcription start sites (TSSs) are given for the gene TSS, upstream antisense TSS (uaTSS), and downstream antisense TSS (daTSS). (B) Observed antisense transcription in human T47D/A1-2 cells. (Left) Heatmaps of counts of the 5’ ends of Start-seq reads centered on gene TSS, uaTSS, and daTSS positions and sorted by chromosome and position. (Middle and right) Heatmaps of counts for the 5’ ends of Start-seq reads and fragment centers of Pol II ChIP-seq reads. Heatmaps are centered on gene TSS positions and sorted by TSS-uaTSS or TSS-daTSS distance. Only gene TSS positions with called uaTSS or daTSS positions, respectively, are included on distance-sorted heatmaps.

Observable genes with antisense transcription display overlap between identified TSSs and the 5´ end of Start-seq reads on both sense and antisense strands (in black and in red; Fig 1A and 1B, respectively). Identified gene TSSs are consistent with RNA-seq read density, and both gene TSSs and antisense TSSs display overlap with Pol II ChIP-seq reads (in blue and red, respectively; Fig 1A). Over 10,391 observed gene TSSs, 5,519 uaTSSs (53% of gene TSSs) and 2,956 daTSSs (28%) were identified (Fig 1B). Over all observed gene TSSs, both a uaTSS and a daTSS were identified at 1,815 genes, indicating a statistically significant association between the two events and implying potential cooperation between upstream and downstream antisense transcription (two-sided Fisher’s exact test: p-value < 10^−6^). To ensure reproducibility, a biological replicate was prepared from the same cell-line, and similar read densities were found at the identified TSSs (S1A Fig). The overall rate of identification is consistent with previous approaches using other experimental methods [7,11,15]. Start-seq read counts are greatest at gene TSSs, with lower counts at uaTSSs and daTSSs (averages of 776, 467, and 276 reads at gene TSSs, uaTSSs, and daTSSs, respectively). Given that the same read threshold was used to call all classes of TSS and that stringent filtering was used to limit miscalling of antisense TSSs, we anticipate that our rate of identification underestimates the prevalence of antisense transcription. daTSSs were also identified in mouse macrophage cells considering observed gene TSS positions found in previous work [11]. 4,921 daTSSs were identified over 12,229 observed gene TSSs, or roughly 40% of genes (S2 Fig). The difference in the identification rate between human T47D/A1-2 cells (28%) and mouse macrophage cells (40%) may be indicative of variability in the landscape of antisense transcription across organisms and cell types. Heatmaps centered on gene TSSs and sorted by the distance from the observed daTSS show that human T47D/A1-2 and mouse macrophage calls are coincident with enriched Pol II ChIP-seq signal (Fig 1B; S2B Fig), supporting calls as bona fide Pol II-dependent transcription events. There is a lack of stranded RNA-seq coverage immediately downstream of uaTSSs and daTSSs (S3A Fig), indicating that transcripts originating from these sites are not present at steady-state in the cytosol and are short-lived.

daTSSs identified in T47D/A1-2 cells were investigated in other cell lines using other experimental data types associated with nascent transcription (MCF-7, GM12878, K562, HeLa S3, and HEK239T cells; S3B Fig). These experiments include a variety of sequencing-based approaches designed to interrogate different biological phenomenon, including direct Pol II-DNA interactions and nascent Pol II-associated transcription. Despite wide ranging differences in technology and cell line, we found an enrichment of nascent RNA- or Pol II-associated signal at daTSS positions. Given the conservation of daTSSs across cell lines, we leveraged a variety of publically available data from different cell lines to annotate daTSSs. Additionally, though we found that a majority of daTSSs are preserved, some do not display signal in other cell lines (S3C Fig). Of the 2,956 daTSSs identified in T47D/A1-2 cells, 1,985 (67%) and 1,966 (67%) display GRO-cap signal in K546 and GM12878 cells, respectively. We examined these genes to see if they displayed cell-specific characteristics. daTSSs with no GRO-cap signal in either K546 or GM12878 cells and that may be considered T47D/A1-2 specific (605 genes; 21%) show an enrichment in categories associated with breast cancer (“breast or ovarian cancer”: p-value = 1.28 x 10^−5^; “mammary tumor”: p-value = 2.10 x 10^−5^). This enrichment suggests cell-line specificity in downstream antisense transcription.

### daTSSs are coincident with promoter-associated sequence features and motifs

**Fig 2:**
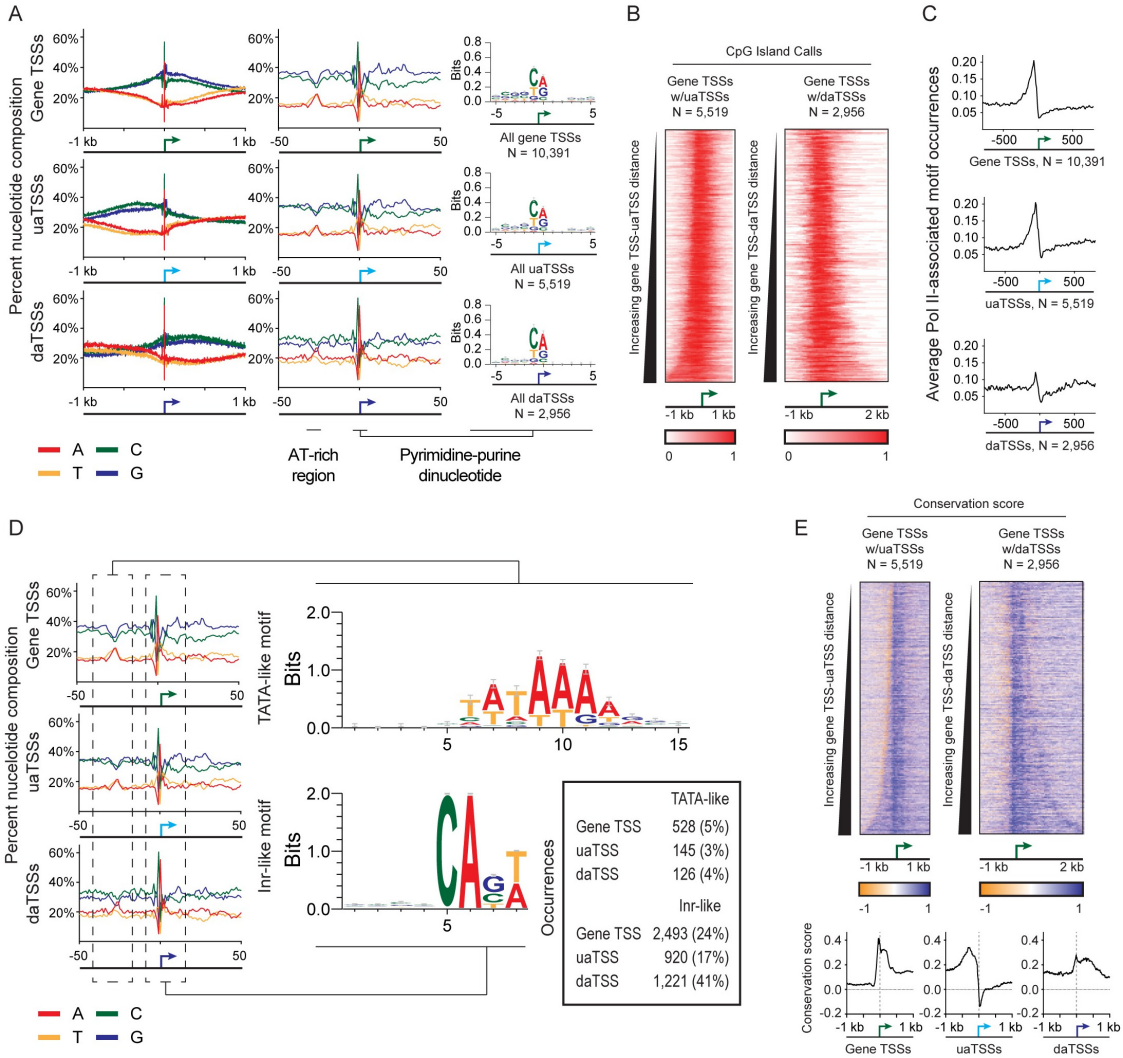
Sequence content is consistent across identified transcription start sites (TSSs). (A) Sequence composition at identified transcription start sites. Nucleotide composition at observed gene TSSs, upstream antisense TSSs (uaTSSs), and downstream antisense TSSs (daTSSs) is shown over +/- 1kb and +/- 50bp windows. Logo plots of sequence within a +/- 5 nt window about TSS positions show a pyrimidine-purine dinucleotide reminiscent of Initiator-binding motifs. (B) Heatmaps of CpG island occurrences about observed gene TSSs sorted by TSS-uaTSS or TSS-daTSS distances. (C) Plots of average occurrences of Pol II-associated sequence motifs in a +/- 1kb window about TSSs. Motif position weight matrices were taken from the Pol II subset of the JASPAR database [18]. (D) Motifs identified by *de novo* discovery near each class of TSS. Motifs were found at (1) an AT-rich region found upstream of TSSs and (2) a small window centered on TSSs containing a distinct pyrimidine-purine dinucleotide. *De novo* motif discovery results in sequences resembling TATA and Inr-binding motifs. Distribution of identified motifs across TSS classes is shown within an inset table. (E) PhyloP conservation score across observed TSSs [19]. Positive values correspond to enhanced sequence conservation. (Top) Heatmaps of PhyloP scores centered on gene TSS positions and sorted by TSS-uaTSS or TSS-daTSS distance. (Bottom) Average PhyloP conversation scores over all gene TSS, uaTSS, and daTSS calls observing +/-1 kb windows.

We examined sequence content at all observed TSSs to both further characterize identified TSSs as associated with Pol II-dependent transcription events and to compare across the three observed TSS classes. Generally, we find that sequence content is similar across all three classes. All TSSs show enrichment for GC content (Fig 2A). Consistent with previous observations, we find enriched GC content upstream of T47D/A1-2 uaTSSs [11]. In addition, we see enriched GC content downstream of daTSSs. Taken together, the antisense TSSs coincide with apparent boundaries of GC content enrichment. The positioning of CpG islands is generally consistent with this observation. Observed uaTSSs and daTSSs broadly coincide with the upstream and downstream edges of promoter-associated CpG islands, respectively (Fig 2B) [17]. However, the alignment between CpG island boundaries of daTSSs is not absolute. Of those 2,392 daTSSs whose associated gene TSS overlaps a CpG island, 1,325 (55%) are within 250 bp of the downstream edge of the CpG island. Thus, observation of a daTSS is not dependent on the presence and relative location of a promoter-proximal CpG island.

Considering a narrow 100-bp window around each TSS (Fig 2A), we observe sequence patterns that are preserved across each class of TSS. Roughly 25 bps upstream of the TSS, we identify an area enriched for TA content. Centered on the TSS itself, there is a distinct sequence pattern with pyrimidine-purine dinucleotide at its center. *De novo* motif discovery reveals enriched sequences similar to TATA box and Inr binding motifs at these regions (Fig 2D) [20]. Though these motifs are present near each class of TSS, subtle differences in relative enrichment of Inr-like motifs (found at 24%, 17%, and 41% of gene TSSs, uaTSSs, and daTSSs, respectively; Fig 2D) may reflect differences in regulation. The occurrences of these specific motifs are consistent with general patterns found in searches performed with Pol II-associated sequence motifs (Fig 2C). Regardless of TSS class, an enrichment of Pol II-associated motifs [18] is observed upstream of TSS positions. GC-box occurrences dominate the enriched motifs (S3D Fig) with fewer motifs identified at daTSSs.

We additionally investigated sequence conservation at daTSS positions. Over promoters displaying antisense transcription, we examined PhyloP conservation scores calculated from sequence alignments across placental mammals [19]. Positive PhyloP scores indicate enhanced sequence conservation immediately upstream of daTSSs (Fig 2E). This is consistent with observations seen at both gene TSSs and uaTSSs [11]. Positive scores are also evident between gene TSSs and daTSSs, implying sequence conservation in this region (Fig 2E). Evolutionary pressure to maintain sequence at daTSSs suggests functional significance for downstream antisense transcription.

### Downstream antisense transcription is not a mark of lowly expressed genes

**Fig 3:**
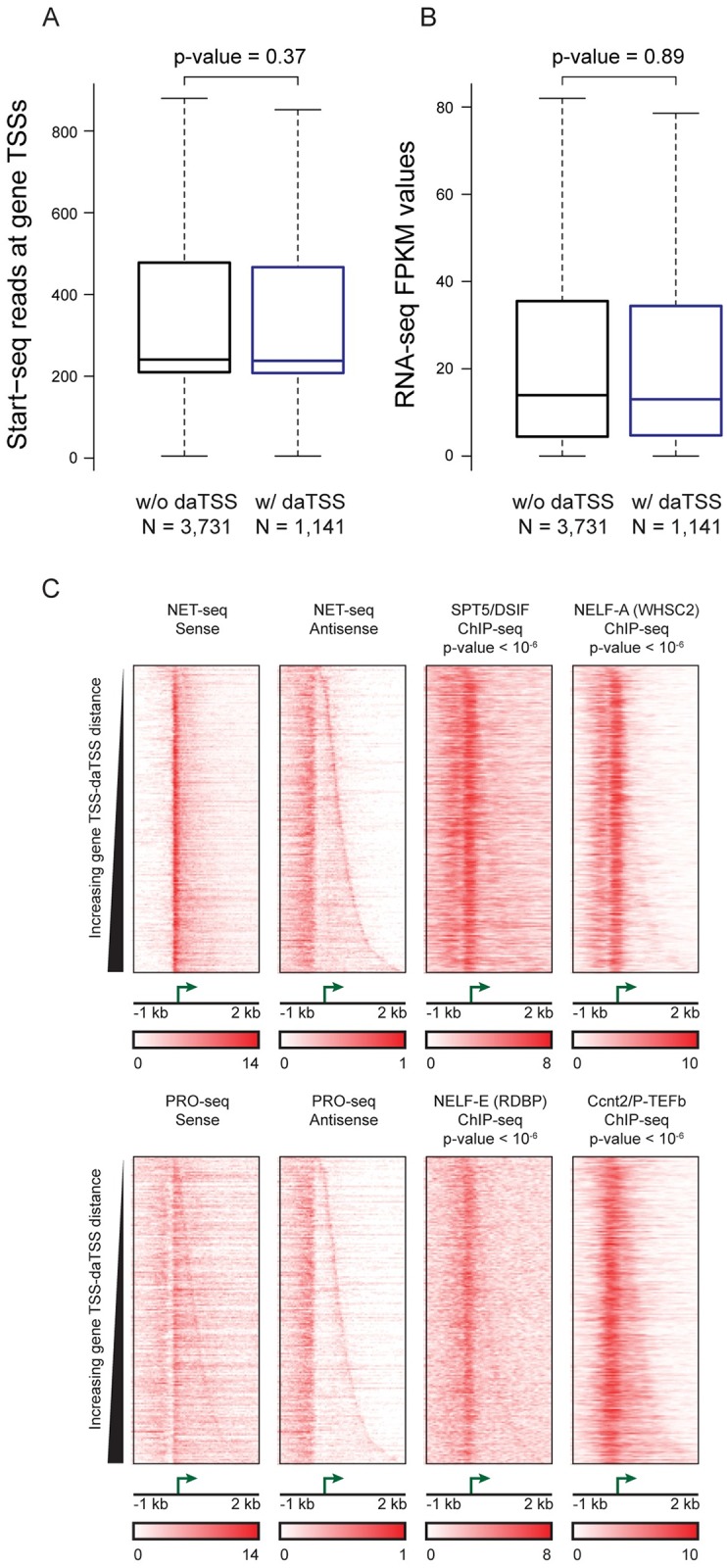
Genes with observed daTSSs show no evidence of enhanced or repressed expression. (A) Box plots of Start-seq read counts at observed gene TSSs for genes with and without daTSSs. Counts consider the overlap of TSS positions of the 5´ end of Start-seq reads. Genes were limited to those without observed uaTSSs. The difference between groups was statistically assessed using Wilcoxon test. (B) Box plots of RNA-seq FPKM values for genes with and without daTSSs. Genes were limited to those without observed uaTSSs. The difference between groups was statistically assessed using Wilcoxon test. (C) Characterization of Pol II and factors involved in Pol II pausing and elongation. Heatmaps are shown centered on observed gene TSSs and sorted by gene TSS-daTSS distance. Pol II association is shown by NET-seq and PRO-seq read density in HeLa and K562 cells, respectively, observing strand specificity and gene orientation. Binding of factors associated with pausing and subsequent elongation is shown by ChIP-seq read density. Displayed p-values were calculated by comparing signal density at daTSSs with equivalent positions at genes without daTSSs by Wilcoxon test (p-values are compiled in S2 Table). ChIP-seq experiments were performed in the following cell lines: HeLa: SPT5/DSIF and NELF-A (WHSC2); K562: NELF-E (RDBP) and Ccn2/P-TEFb).

We next sought to examine the connection between downstream antisense transcription and the expression of associated genes. Recent studies have proposed that promoter-proximal downstream antisense transcription represses expression of upstream genes [15]. We examined the expression of genes displaying downstream antisense transcription in two distinct ways. We first compared the extent of transcription initiation at those genes with and without observed daTSSs (categories “w/ daTSSs” and “w/o daTSSs”; Fig 3A). We restricted this comparison to those genes without observed uaTSSs to limit the effect of upstream antisense transcription on observed trends (N = 4,872). The difference between genes with and without daTSSs is not statistically significant (Wilcoxon test: p-value = 0.37). We also find that the extent of downstream antisense transcription as measured by Start-seq read counts at daTSS positions shows no correlation with read counts at gene TSSs (genes with no observed uaTSSs; Spearman test: rho = 0.017, p-value = 0.56).

We next compared steady-state transcript levels using RNA-seq-derived FPKM values. The results for RNA-seq analysis are comparable to those derived from Start-seq (Fig 3B). Again, comparisons were restricted to genes without observed uaTSSs. The difference in FPKM values between genes with and without daTSSs is not statistically significant (Wilcoxon test: p-value = 0.89). In summation, downstream antisense transcription is not associated with lowly expressed genes in human T47D/A1-2 cells. These results suggest convergent transcription near gene promoters is not inhibitory.

These results are distinct from those reported in recent a study describing promoter-proximal downstream antisense transcription as mark of lowly expressed genes [15]. This conclusion was based upon differences in the gene body density of Net-Seq reads between genes displaying only upstream antisense or downstream antisense transcription. When we use a similar categorical separation of genes, we find that genes displaying only uaTSSs show greater steady-state transcription levels than genes displaying only daTSSs (Wilcoxon and Kolmogorov-Smirnov tests: p-values < 10^−6^; S4A Fig). However, these differences appear attributable not to the presence of downstream antisense transcription but to the absence of upstream antisense transcription. Considering genes without daTSSs (N = 7,435), those genes with uaTSSs display significantly higher steady-state transcription than genes without uaTSSs (Wilcoxon test: p-value < 10^−6^; S4B Fig). These results suggest that a complete understanding of the effects of downstream antisense transcription requires deconvolution from that of upstream antisense transcription.

An examination of promoter proximal pausing suggests that downstream antisense transcription may be coordinated with transcription of associated genes. We find that Ccnt2 (or CycT2), part of the P-TEFb complex involved in the regulation of Pol II elongation, selectively associates to the area between gene TSSs and daTSSs and shows statistically significant enrichment compared to equivalent regions at genes without daTSSs (Wilcoxon test, p-value <10^−6^; Fig 3C). This is coincident with enrichment of components of NELF and DSIF (Wilcoxon tests, p-values < 10^−6^; Fig 3C). PRO-seq experiments measuring strand-specific association of elongating Pol II also show a sense-strand enrichment of Pol II near the daTSS (Fig 3C), further supporting a connection between Pol II pausing and antisense transcription. These results do not necessarily imply that genes displaying downstream antisense transcription are more paused. Pausing itself is a highly regulated event in gene transcription with connections to signal-dependent gene expression [21]. Pol II-dependent transcription antisense of genes may contribute to signal-dependent response of paused genes. Consequently, while downstream antisense transcription does not appear to correlate with steady-state transcription levels, antisense transcription could affect signal-dependent gene expression changes.

### daTSSs respect nucleosome positioning and coincide with promoter-associated histone marks

**Fig 4:**
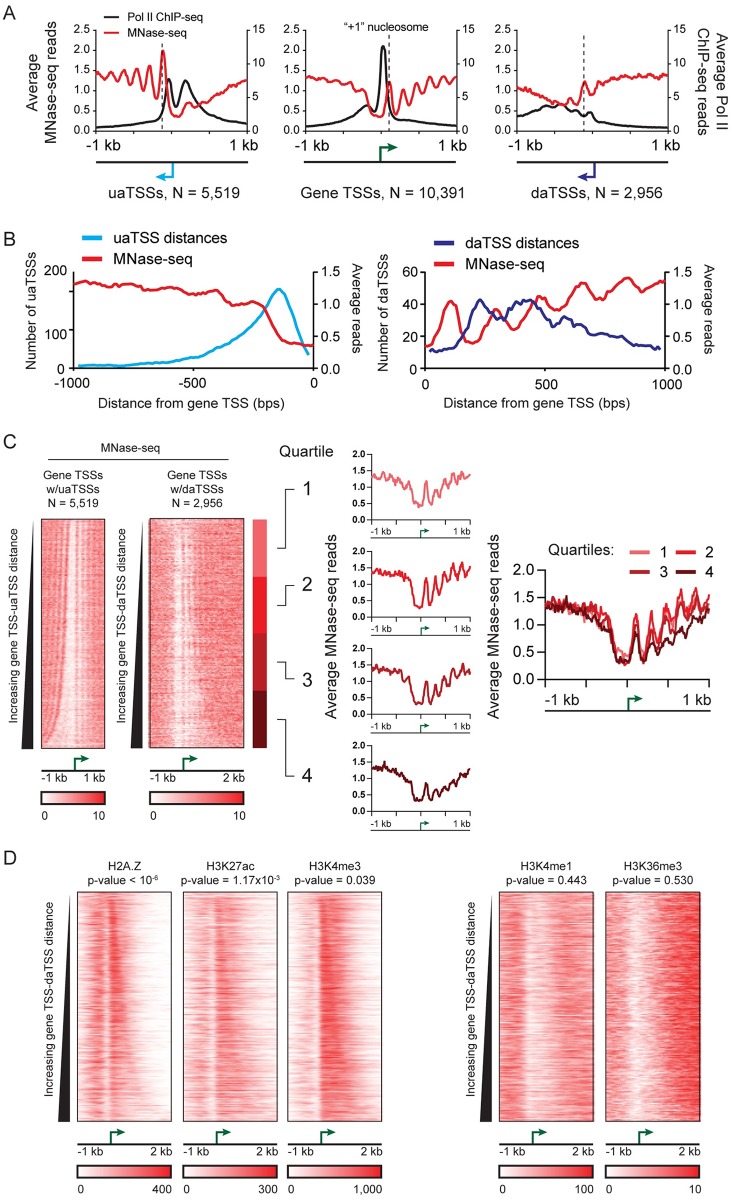
Nucleosomes show regular positioning at downstream antisense TSSs (daTSSs) and comprise a promoter-specific chromatin environment. (A) Average read densities compared for Pol II ChIP-seq (black) and MNase-seq experiments (red). Plots are centered on uaTSS, gene TSS, and daTSS positions. “+1” nucleosome positions are indicated by dashed lines. (B) Histograms of gene TSS-uaTSS and TSS-daTSS distances (cyan and blue, respectively) compared with average MNase-seq density at gene TSSs (red). Negative and positive distance measurements correspond to regions upstream and downstream, respectively, of gene TSS positions. (C) MNase-seq read densities at observed TSSs. (Left) Heatmaps of MNase-seq read densities centered on gene TSSs and sorted by TSS-uaTSS or TSS-daTSS distance. (Right) MNase-seq densities for genes quartiled by gene TSS-daTSS distance. Quartiles are indicated on the heatmap of MNase-seq read density sorted by gene TSS-daTSS distance. Plots of average MNase-seq read densities are shown individually and overlaid for each quartile. (D) Heatmaps of ChIP-seq read density of histone modifications from HMECs showing a distinct chromatin environment between gene TSSs and daTSSs enriched for certain promoter-associated histone marks. H2A.Z histones and H3K4me3 and H3K27AC modifications are associated with active promoter regions while H3K4me1 and H3K36me3 modifications are associated with enhancers and transcription elongation within gene bodies, respectively. Displayed p-values were calculated by comparing signal density at daTSSs with equivalent positions at genes without daTSSs by Wilcoxon test (p-values are compiled in S2 Table). Heatmaps and plots of average ChIP-seq density for both gene TSS-uaTSS and gene TSS-daTSS pairs are given in S5 Fig.

We find that the daTSS position coincides with the downstream edge of a chromatin environment displaying promoter-associated features. To investigate the interplay between antisense transcription and nucleosome positioning, we performed MNase-seq on T47D/A1-2 cells. As evidenced in the resulting data, nucleosomes are regularly positioned relative to all three classes of identified TSSs. Like gene TSSs, MNase-seq read density is consistent with “+1” nucleosomes placed immediately downstream of daTSSs and uaTSSs, though MNase-seq peaks are less sharp when centered on daTSS positions (Fig 4A). The daTSS location respects the regular positioning of promoter-proximal nucleosomes. A histogram of gene TSS-daTSS distances is anti-correlated with average MNase-seq density (Fig 4B), implying that daTSSs fall between regularly-spaced nucleosomes oriented at gene TSSs. This pattern is reproduced in MNase-seq data from other breast epithelial cells (MCF-7 cells; S1B Fig) [22]. The observed MNase-seq density is consistent with Pol II ChIP-seq density at daTSS positions (Fig 4A). Downstream of observed daTSS positions, we find a regular pattern of Pol II ChIP-seq read density that is attributable to Pol II initiating at gene TSSs and that mirrors the MNase-seq data. As previously described [11], we find that regions between gene TSSs and associated uaTSSs are nucleosome depleted. This is clear in heatmaps of MNase-seq density (Fig 4C, left). In comparison, the region between gene TSSs and daTSSs displays a regular pattern of positioned nucleosomes (Fig 4C, left). When quartiled by TSS-daTSS distance, TSSs with greater distances show less distinct downstream MNase-seq peaks, perhaps indicating less regular or more transient nucleosome associations at these positions (Fig 4C, right). However, the location of MNase-seq peaks downstream of gene TSSs seem to be similar across genes with and without daTSSs (S4C Fig), indicating that the location of the gene TSS predominantly influences nucleosome positioning.

Given that nucleosomes are regularly positioned at identified TSSs, we sought to characterize histone modifications in those regions. We find that histone modifications at those nucleosomes positioned between the gene TSS and daTSS are distinct from proximal regions. ChIP-seq data in HMEC cells [23] show an enrichment of histone marks associated with active promoters. When compared to equivalent positions at genes without daTSSs, H3K27ac and H3K3me3 modifications show significant enrichment by Wilcoxon test (p-values of 1.17 x 10^−3^ and 3.94 x 10^−2^ for H3K27ac and H3K4me3 modifications, respectively; Fig 4D). There is tendency for histone modification enrichment to end at the daTSS, as clearly seen in profiles for histone variant H2A.Z (Wilcoxon test: p-value < 10^−6^; Fig 4D). This same enrichment is not seen for H3K4me1 and H3K36me3 modifications, associated with enhancers and actively transcribed regions, respectively (Wilcoxon test: p-values of 0.53 and 0.44, respectively). Consistent with the lack of nucleosomes in this region, we do not see the same enrichment between the gene TSS and uaTSS (S5 Fig).

### Transcription factors and chromatin remodeling machinery associate with an open genomic region at daTSSs

**Fig 5:**
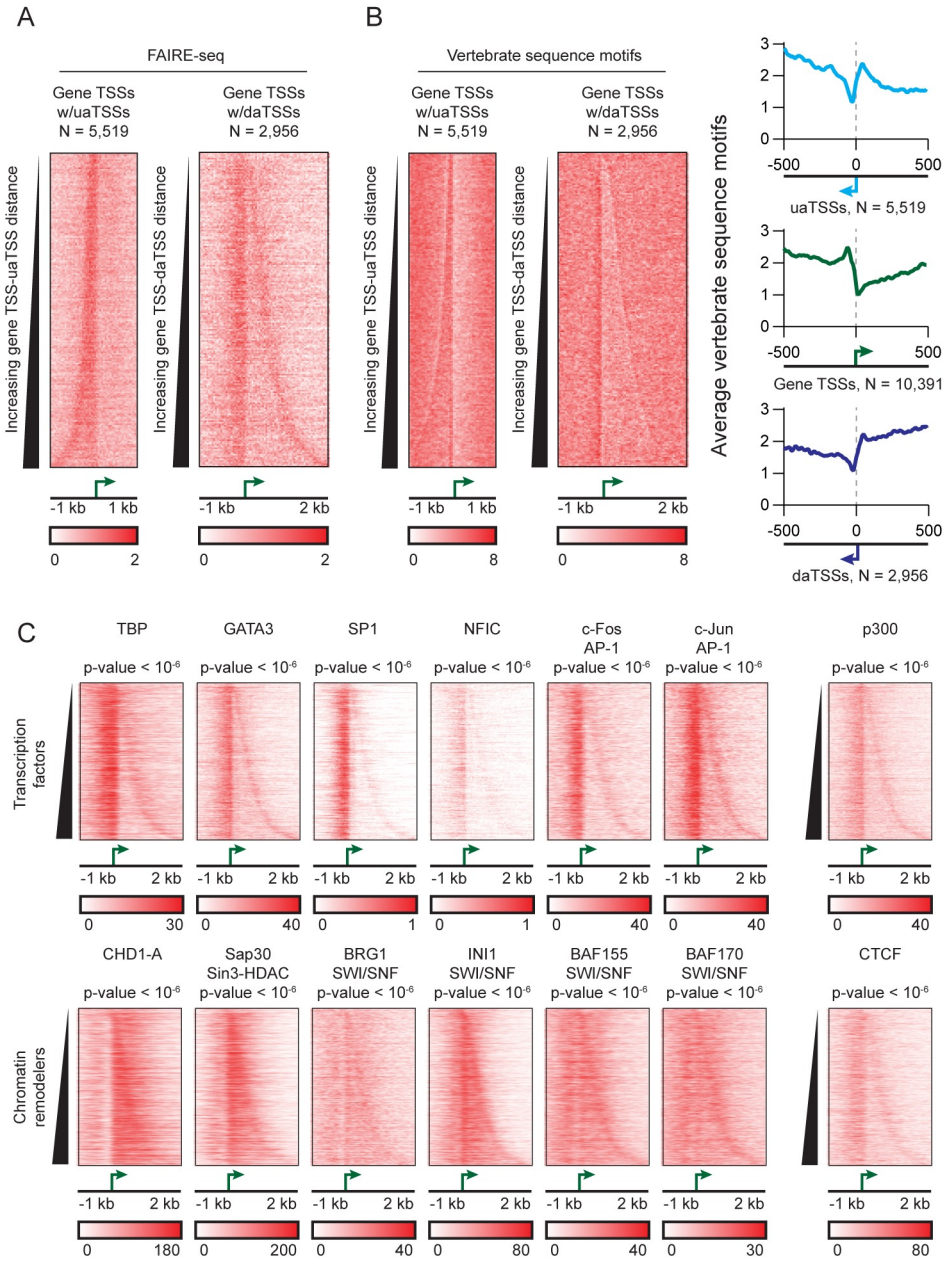
Binding of trans-regulatory factors is coincident with an open DNA region at downstream antisense TSSs (daTSSs). (A) Heatmaps and plots of FAIRE-seq read density over identified TSS positions. Heatmaps of FAIRE-seq density are centered on gene TSSs and sorted by gene TSS-uaTSS or gene TSS-daTSS distances. Areas with enriched signal correspond to open DNA regions. (B) Occurrences of vertebrate sequence motifs over identified TSS positions. (Left) Heatmaps of motif occurrences centered on gene TSSs and sorted by TSS-uaTSS or TSS-daTSS distance. (Right) Plots of the average number of motif occurrences over each class of TSS. Motif position weight matrices were taken from the JASPAR database [18]. (C) Heatmaps of ChIP-seq read density for a variety of trans-regulatory factors across heterologous cells lines. Transcription factors and chromatin remodelers display distinct modes of binding relative to gene TSS and daTSS positions. ChIP-seq experiments were performed in the following cell lines: GM12878: TBP and NFIC; K562: c-Fos, c-Jun, CHD1-A, and Sap30; MCF7: GATA3 and p300; A549: SP1 and CTCF; HeLa: BRG1, INI1, BAF155, and BAF170. Displayed p-values were calculated by comparing signal density at daTSSs with equivalent positions at genes without daTSSs by Wilcoxon test (p-values are compiled in S2 Table).

Given the observed histone modification profile, we next sought to characterize the relationship between the association of transregulatory factors and antisense transcription at gene promoters. The association of transcription factors is enriched at open regions of DNA. We characterized accessible regions of DNA in T47D/A1-2 cells using FAIRE-seq. Like gene TSSs and uaTSSs, FAIRE-seq reveals an open genomic region at daTSS positions (Fig 5A) [24]. This observed density is consistent with FAIRE-seq results reported in a previous study (S1C Fig) [24] and recapitulates observations made using DNase-seq [15]. Following characterization by FAIRE-seq, we performed protein motif searches in similar areas to characterize the potential of these areas to interact with DNA-binding proteins. Analysis of known vertebrate motif occurrences shows a depletion of protein-binding motifs between gene TSSs and daTSSs and an enrichment of motifs immediately upstream of daTSSs (Fig 5B) [18]. Consistent with these areas being open and enriched for protein-binding motifs, ChIP-seq data [23] reveal that the daTSS coincides with the binding of trans-regulatory factors. Transcription factors were found to associate with open regions at both the gene TSS and the daTSS (Fig 5C, top). Comparisons with equivalent positions at genes without daTSSs show a significant enrichment of transcription factor-associated signal at daTSS positions (Wilcoxon test: p-values < 10^−6^; Fig 5C, top, and S2 Table). We present selected transcription factors known to associate at gene promoters and broadly participate in a number of signal-dependent pathways. However, the coincidence of p300, a known co-activator of numerous additional transcription factors [25], implies potential interaction with many others at daTSS positions (Fig 5C, top-right). In contrast, chromatin remodelers were found to associate in the area between the gene TSS and the daTSS with a significant enrichment of associated signal at daTSS positions (Wilcoxon test: p-values < 10^−6^; Fig 5C, bottom, and S2 Table). Though this area displays high GC content, this alone does not explain the enrichment of chromatin remodelers; randomly selected genomic regions matched for GC content show diminished signal relative to these regions (Wilcoxon test: p-value < 10^−6^). Like daTSSs, a variety of trans-regulatory factors associate to uaTSS positions (S6, S7 and S8 Figs). However, differences in regions between gene and antisense TSSs distinguish these two classes of TSS. Unlike daTSSs, transcription factors associate to the nucleosome-depleted areas between gene TSSs and uaTSSs while chromatin remodelers do not. This likely reflects differences in nucleosome occupancy between nucleosome-rich TSS-daTSS regions and nucleosome-deprived TSS-uaTSS regions (Fig 4). Factors involved in the deposition of histone marks (CHD1-A and Sap30) and in the positioning of nucleosomes (SWI/SNF-associated factors) may contribute to the distinct chromatin environment seen at this region, with CTCF potentially contributing to the definition of this region (Fig 5C, bottom-right). This suggests a mechanism similar to that observed in yeast where antisense transcription may contribute to a chromatin environment that ultimately impacts gene expression [26].

## Discussion

Our analyses indicate that downstream antisense transcription proximal to gene promoters is common in mammals. Its coincidence with a number of different regulatory features suggests that antisense transcription borders the chromatin environment characteristic of promoters and may possess a regulatory role. Previous studies have characterized convergent transcription as a repressive feature of genes [27]. The promoter-proximal convergent transcription described here is a narrow subset of convergent transcription, where downstream antisense transcription initiates at or within 2 kb of a gene TSS. Based on our analysis, we see little evidence for repression of associated genes by downstream antisense transcription. Considered categorically, comparisons between all genes and those displaying observable daTSSs fail to show significant differences in levels of transcription initiation and steady-state expression levels (Fig 3). Ultimately, the interplay between antisense transcription and gene expression will be complex, as coincidence of daTSSs positions with other promoter-associated features suggests interplay with other regulatory pathways (S9 Fig).

Active enhancers are often present and transcribed within intronic regions of gene bodies. Given the position of daTSSs downstream of gene TSSs, there is a question as to whether downstream antisense transcription is predominantly the consequence of canonical enhancer activity within gene introns. The lack of a direct connection to increased expression levels suggests that downstream antisense transcription is not associated with active enhancers regulating the nearby gene. Though a majority of daTSSs are positioned within introns (74%), a similar proportion remain overlapped with introns when gene models are randomly shuffled (75%), indicating that daTSSs are not significantly enriched within introns (S3 Table). There is also a conspicuous lack of signal attributable to enhancer-associated H3K4me1 at observed daTSSs, though there is an apparent association of p300 and H3K27ac marks (Figs 4D and 5C).

**Fig 6:**
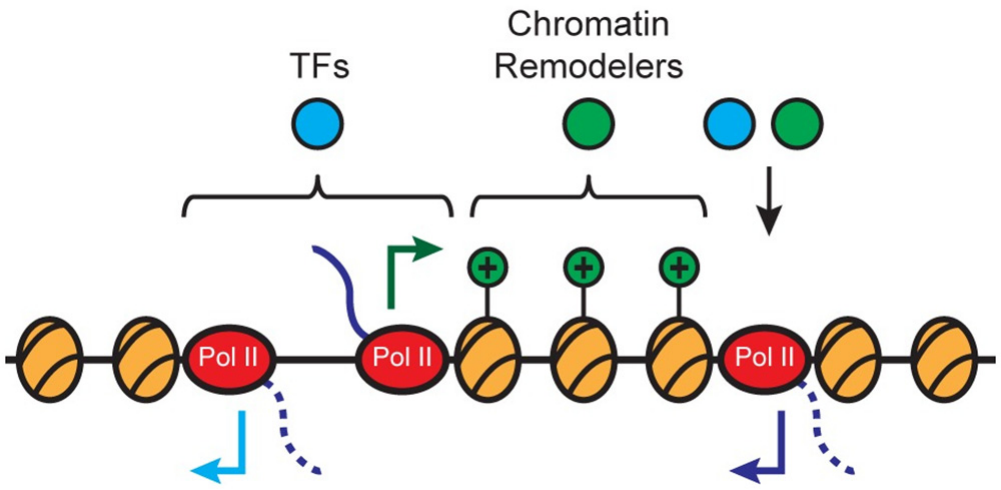
Antisense transcription correlates with promoter-associated features. Nucleosomes display regular positioning relative to both sense and antisense TSSs, with a depleted region existing between gene TSSs and uaTSSs. Nucleosomes between gene TSS and daTSS positions display histone marks associated with active promoters. Transregulatory factors display categorically distinct binding, with transcription factors associating in the area between the TSS and uaTSS and with chromatin remodelers associating in the area between the gene TSS and daTSS.

Rather than describing a functionally distinct element, e.g. proximal enhancers, downstream antisense transcription seems to be a feature of promoters themselves. Along with antisense transcription upstream of gene TSSs, downstream antisense transcription may be an intrinsic feature at many mammalian promoters (Fig 6). There appears to be a connection between antisense transcription and promoter-specific features at genetic and epigenetic levels (for an overview of features at each class of TSS, see S9 Fig). daTSSs respect the positioning of promoter-proximal nucleosomes, with observed daTSSs falling within valleys of MNase-seq read density (Fig 4B). Downstream antisense transcription may affect nucleosome organization at promoters; genes with larger distances between gene TSSs and daTSSs display less prominent nucleosome-associated peaks in MNase-seq data (Fig 4C). daTSSs also coincide with the binding of transregulatory factors. In particular, regions between gene TSSs and daTSSs show association of chromatin remodeling factors (Fig 5C). These factors potentially contribute to the chromatin environment bordered by gene TSSs and daTSSs and distinguished by enrichment of promoter-associated histone marks. It is not clear whether these functions are attributable to generated transcripts or transcription itself, though produced transcripts are not apparently stable (S3A Fig). Recent studies suggest that non-coding RNAs generated near promoters participate in the establishment of nucleosome occupancy [28].

Though both are proximal to promoters, daTSSs and uaTSSs exist in distinct epigenetic environments (S9 Fig). daTSSs and uaTSSs display fundamentally different relationships with nucleosomes in the promoter region. uaTSSs are an apparent boundary of the nucleosome depleted region at promoters [11] while daTSSs initiate from between regularly oriented nucleosomes downstream of gene TSSs (Fig 4). The different relationships with nucleosomes seem to inform in part the differences observed with other epigenetic features. Nucleosome depletion near uaTSSs allows for transcription factor association between uaTSSs and gene TSSs while the presence of nucleosomes likely prevents transcription factor association between daTSSs and gene TSSs (Fig 5; S6 Fig) [11]. Likewise, the presence of nucleosomes gives functional relevance to the association of chromatin remodelers observed between daTSSs and gene TSSs but not between uaTSSs and gene TSSs (Fig 5; S7 Fig).

Despite differences in epigenetic features, tendency for association with transregulatory factors, and capacity to produce stable RNA transcripts, all three classes of TSS described in this work display similarities in sequence content, including enrichment for GC content and Pol II-associated sequence motifs (Fig 2). As such, antisense transcription appears to be encoded in genetic sequence. This connection between sequence content and epigenetic features provides the compelling suggestion that antisense transcription encoded by sequence may direct the positioning of nucleosomes and deposition of histone marks. Antisense transcription may also participate in signal-dependent modulation of epigenetic content where activation of sequence-encoded antisense TSS precedes nearby changes in chromatin structure. In this way, the collection of transcription initiation-associated sequence motifs near promoters may define regulatory potential for a given gene. This connection to sequence also provides a means to interrogate antisense transcription function. Future studies with selective mutation of associated sequence motifs may elucidate the function of antisense transcription and its coincidence with promoter-associated features. Directed mutagenesis could also establish the extent of the effect of antisense transcription on the chromatin environment at promoters.

We characterized downstream antisense transcription initiating near gene promoters in human T47D/A1-2 cells. daTSSs fall between regularly positioned nucleosomes downstream of gene TSSs. Histones within this region are enriched for marks closely associated with active promoter regions, such as H3K4me3 and H3K27ac modifications. Chromatin remodeling complexes show enriched binding upstream of observed daTSS positions, suggesting that antisense transcription contributes to the establishment and maintenance of a promoter-specific chromatin environment. Downstream antisense transcription is common to many human promoters, and daTSSs correlate with the downstream edge of promoter-associated chromatin features. Coincidence of daTSSs with these features suggests interplay between antisense transcription and regulatory pathways.

## Materials and Methods

### Collection and processing of Start-seq data

T47D/A1-2 cells were cultured in DMEM containing 10% FBS. Prior to RNA isolation, cells were cultured in medium supplemented with 5% charcoal dextran-treated serum for at least 24 hours. The A1-2 cell line is a previously described derivative of the T47D breast cancer cell line that overexpresses rat glucocorticoid receptor (GR) and contains a stably-integrated MMTV luciferase reporter gene [29]. Short capped RNA was isolated from T47D/A1-2 cells as previously described [16]. Libraries were generated using the Illumina TruSeq small RNA kit. Two independent replicates were performed. A primary dataset was generated from combined Illumina HiSeq and MiSeq runs. This data set was used in all TSS calling and downstream analysis. A secondary validation data set with fewer reads was generated on an Illumina MiSeq run. This data set was used to validate reproducibility of read density at called TSSs (S1A Fig). For this and other in-house sequencing experiments, libraries were prepared and sequenced by the NIH Intramural Sequencing Center (NISC).

Start-seq reads were first filtered by quality score; reads with an average Sanger score less than 20 were removed from analysis. Following quality filtering, Cutadapt (version 1.2.1) was used to remove adapter sequences [30]. Alignment of Start-seq reads was performed using Bowtie (version 0.12.8) to hg19 or mm9 genome assemblies [31]. From each uniquely mapped Start-seq fragment, the 5’ end was taken forward into the TSS calling procedure.

### Identification of TSSs

Identification of TSSs was performed based on methods described previously [11,16], TSS identification was guided by RefSeq annotation (retrieved 05/09/2014) [32]. From the reference annotation, a list of non-redundant TSSs was taken from all mRNA RefSeq IDs (“NM_”). 2000-nt search windows were created about each RefSeq TSS. If search windows overlapped and had the same common gene name, those search windows were merged. For other overlapping search windows, boundaries were defined as the midpoint between associated TSSs. The intersection of search windows and 5’ Start-seq ends was then determined.

TSSs were called within each window in which the strand-specific 5’ end read count at any given nucleotide position met or exceeded a threshold of 5 reads. This threshold was determined in a previously described method [33]. In short, the FDR was estimated based upon the distribution of Start-seq reads across the genome and a background model where the probability of finding a given number of aligned reads by chance is given by a sum of Poisson probabilities. The read threshold was selected to allow less than 1 expected false positive by this measure.

In those windows where a single nucleotide position met or exceeded the read threshold, a gene TSS was called. The calling method aims to select as the TSS the position with the highest read counts in window region with the highest read density. To accomplish this, two potential TSSs were first determined. The first was the position with the most aligned 5’ ends across the entire window. For the second, the search window was divided into 200-nt bins at every 10 nt across the search window. At the 200-nt bin with the most overlapped 5’ ends, the second potential TSS was called as the position with the most aligned 5’ ends. Of the two putative sites, the TSS closest to the associated annotated RefSeq TSS was selected.

Following gene TSS identification, uaTSSs and daTSSs were found. For uaTSSs and daTSSs, search spaces were defined in antisense orientation as 1 to 1000 nt upstream and 1 to 2000 nt downstream, respectively. uaTSSs and daTSSs were called at the position with the most aligned 5’ ends within the search window if the count at any single nucleotide position met or exceeded a threshold of 5 reads. To ensure that identified daTSSs were not simply mis-called gene TSSs or uaTSSs, additional criteria were used to filter daTSS calls. A daTSS call was filtered if (1) within 1000 nt upstream of the daTSS there was a genomic position with Start-seq reads greater than or equal to 10% of reads at the associated gene TSS on the same strand as the gene TSS, implicating the daTSS as a potential uaTSS for an uncalled gene TSS or (2) it was within 1000 nt of an annotated TSS on the same strand, implicating the daTSS as a potential gene TSS.

### Processing of MCF7 ChIP-seq data

Prior to alignment, Pol II ChIP-seq reads from MCF-7 cells [23] (see also Supplemental Table 1) were filtered based on quality; reads with an average Sanger quality score less than 20 were removed from analysis. Following quality filtering, Cutadapt was used to remove adapter sequences [30]. Alignment was performed using Bowtie [31]. Fragment lengths were estimated using Homer (version 4.6) [34]. From each uniquely mapped fragment, the fragment was extended based on the estimated length, and the fragment center was subsequently found.

### Analysis of sequence content

Considering each TSS group separately, the nucleotide composition of each position in a -1000 to +999 window was determined and reported as a percentage. Logo plots were generated using Web Logo 3 considering sequences in a -5 to +5 window about identified TSSs [35].

To identify occurrences of Pol II-associated and known vertebrate motifs, FIMO (version 4.10.0) was used considering a p < 0.0001 significance cutoff and a 0-order Hidden Markov Model from promoter regions as background [36]. Publically available position weight matrices from JASPAR were used in motif identification [18]. The JASPAR POLII database (2008 version; 13 motifs) and vertebrate motifs in the JASPAR CORE database (2014 version; 205 motifs) were used for Pol II-associated and known vertebrate motif identification, respectively. Across all promoter regions, on the order of 10^5^ Pol II-associated and 10^7^ known vertebrate motifs were identified. Motifs and additional information are available at http://jaspar.genereg.net/.

*De novo* motif discovery was performed using MEME (version 4.10.0) with default parameters [37]. Sequence windows from -35 to -20 and from -5 to +5 relative to TSS positions were used. Sequences from each TSS class were combined prior to motif analysis. Logo plots were generated using Web Logo 3 after aligning identified motifs [35].

CpG island heatmaps reflect the intersection of annotated CpG islands retrieved from UCSC Genome Browser [17] with TSS-centered windows.

Sequence conservation heatmaps were generated using phyloP scores from placental mammal alignments retrieved from UCSC Genome Browser [19]. Each position in the heatmap represents the average score over all positions in a 40-bp bin for which phyloP scores were available.

### Functional enrichment of T47D/A1-2-specific daTSSs

GRO-cap data from K562 and GM12878 cells [7] were compared to daTSSs identified in T47D/A1-2 cells. If a given site did not have GRO-cap signal in either K562 or GM12878 cells, that daTSS was considered T47D/A1-2 specific. The list of genes with T47D/A1-2-specific daTSSs was applied to Ingenuity Pathway Analysis [38] considering experimentally observed associations over mammalian tissues and cell lines.

### Collection and analysis of MNase-seq data

Nuclei were harvested from cultured T47D/A1-2 cells and digested for 5 minutes at 37°C with a range of MNase (Worthington) concentrations. Reactions were stopped by the addition of EDTA and then treated with RNase and proteinase K. Digested DNA was isolated by phenol/chloroform extraction and ethanol precipitation. Libraries were prepared using an Illumina TruSeq sample preparation kit and sequenced on an Illumina HiSeq for paired-end 50 base reads.

Publically available MNase-seq data [22] and data generated in this work were prepared in the same way. Prior to alignment, MNase-seq reads were filtered based on quality; reads with an average Sanger quality score less than 20 were removed from analysis. Following quality filtering, Cutadapt was used to remove adapter sequences [30]. Alignment of MNase-seq read pairs was performed using Bowtie [31]. From each uniquely mapped fragment, the fragment center was found. Any report of MNase-seq coverage only considers the fragment-center position.

### Analysis of FAIRE-seq data

FAIRE-seq data were collected as described previously [24]. Publically available FAIRE-seq data [24] and data generated in this work were prepared in the same way. Prior to alignment, FAIRE-seq reads were filtered based on quality; reads with an average Sanger quality score less than 20 were removed from analysis. Following quality filtering, Cutadapt was used to remove adapter sequences [30]. Alignment of FAIRE-seq reads was performed using Bowtie [31]. Aligned FAIRE-seq reads were then de-duplicated using Picard (version 1.118) [39]. FAIRE-seq fragment lengths were estimated using Homer [34]. For each uniquely mapped fragment, the fragment was extended based on the estimated length, and the estimated fragment center subsequently found. Reported FAIRE-seq coverage only considers the fragment-center position.

### Collection and analysis of RNA-seq data

Over biological triplicates, total RNA was harvested using an RNeasy Kit (Qiagen) with on-column DNase treatment. RNA quality was validated by Bioanalyzer (Agilent). Paired-end strand-specific poly-A enriched libraries were sequenced on an Illumina HiSeq 2500 for 125 base paired-end reads.

Prior to alignment, RNA-seq reads were filtered based on quality; reads with an average Sanger quality score less than 20 were removed from analysis. Following quality filtering, Cutadapt was used to remove adapter sequences [30]. Insert lengths were estimated by transcriptome alignment using Bowtie [31]. Sequence alignment was then performed using TopHat (version 2.0.4) [40]. Following de-duplication by Picard [39], alignments from individual replicates were merged. FPKM values were calculated using Cufflinks (version 2.2.1) [41].

### Analysis of public data sets

For publically available data sets (with the exceptions of MCF7 Pol II ChIP-seq data and of FAIRE-seq and MNase-seq validation data sets), read coverage files were retrieved from public depositories (S1 Table). Given that reported read densities were considered, the data processing of the original authors was effectively observed. deepTools was used to generate matrices describing the intersection of read coverage with TSS-centered genomic windows observing strand specificity when appropriate [42]. These matrices were then used to generate heatmaps.

### Generation of heatmaps and other plots

Heatmaps consider 40-bp/40-nt bins over TSS-centered windows. Unless otherwise noted, each position in a heatmap gives the number of reads or other features overlapping with that bin. Heatmap images were generated using Partek (version 6.6) [43]. Two-dimensional plots, unless otherwise noted, consider 10-bp/10-nt bins and report average values across all TSSs considered. To test enrichment of ChIP-seq signal at daTSS positions, ChIP-seq coverage was found in a 100-bp window about all identified daTSSs. Equivalent regions were found at genes without daTSSs by selecting a 100-bp window shifted downstream of TSSs by the median observed TSS-daTSS distance (507 nts). The significance of enrichment was then calculated by Wilcoxon test comparing the two groups.

To generate the gene TSS-centered panels in S5, S6, S7 and S8 Figs, uaTSS- and daTSS-centered plots were first reflected across uaTSS and daTSS positions, respectively, to orient these plots relative to gene TSSs. These plots were then translated upstream or downstream by the median distances observed between gene TSSs and uaTSSs or between gene TSSs and daTSSs across calls made in T47D/A1-2 cells.

### Accession numbers

The data sets supporting the results of this article are available in the GEO repository, GSE74308.

## Supporting Information

S1 Table Sources of public data. (PDF)

S2 Table Enrichment test p-values for ChIP-seq data sets. (PDF)

S3 Table Overlap of daTSSs with RefSeq-annotated exons and introns. (PDF)

S1 Fig Validation of study reproducibility. Reported data (red) and validation data (blue) are shown anchored at identified TSS positions in both heatmaps and two-dimensional plots. Validation data are taken from biological replicates or from similar cell lines. Read density of a Start-seq biological replicate (T-47D/A1-2 cells) (A), FAIRE-seq in T-47D/A1-2 cells [24] (B), and of MNase-seq in MCF-7 cells [22] (C) are shown at identified TSS positions.
(TIF)

S2 Fig Identification of transcription start sites in mouse macrophage cells. (A) Observed transcription at gene Rpe. Genomic context is given by RefSeq annotation (green), Pol II ChIP-seq coverage (red), and counts for the 5’ ends of Start-seq reads in both sense and anti-sense orientations (black). Observed transcription start sites (TSSs) are given for the gene TSS, upstream antisense TSS (uaTSS), and downstream antisense TSS (daTSS). (B) Heatmaps of counts for the 5’ ends of Start-seq reads over gene TSS, uaTSS, and daTSS positions. (Right panel) Heatmaps of counts for the 5’ ends of Start-seq reads and fragment centers of Pol II ChIP-seq reads. Heatmaps are centered on gene TSS positions and sorted by gene TSS-uaTSS (left) or gene TSS-daTSS distance (right). Only TSS positions with called uaTSS or daTSS positions, respectively, are included on the heatmaps.
(TIF)

S3 Fig Comparison of called TSS positions across other transcription-associated data. (A) Average RNA-seq coverage in T47D/A1-2 cells across identified TSS positions. (B) Heatmaps of read density from Pol II-associated sequencing approaches. Pol II ChIP-seq, NET-seq, GRO-seq, GRO-cap, and PRO-seq were performed over a variety of cell lines (indicated on figure). Each heatmap is centered on observed gene TSS position and sorted by increasing gene TSS-daTSS distance. (C) Categorization of T47D/A1-2-called daTSSs by presence of GRO-cap signal in heterologous cell lines. daTSSs were placed into a separate category if no GRO-cap signal was found within 10 nt of the observed daTSS. 987 (33%) and 971 (33%) daTSSs called in T47D/A1-2 cells were found to have no significant GRO-cap signal in GM12878 and K562 samples, respectively. (D) Plots of average occurrences of Pol II-associated sequence motifs. Motif occurrences were identified using FIMO [36]. Motif position weight matrices were taken from the Pol II subset of the JASPAR database [18].
(TIF)

S4 Fig Comparison of gene RNA-seq FPKM values and MNase-seq coverage profiles by antisense transcription status. (A) Empirical cumulative distributions of gene RNA-seq FPKM values for genes displaying only daTSSs (blue) and only uaTSSs (cyan). Inset p-value was determined by Kolmogorov-Smirnov test. (B) Box plots of RNA-seq FPKM values for all genes, genes without uaTSSs, and genes without daTSSs. Reported p-values were determined by Kolmgorov-Smirnov tests. (C) Average MNase-seq read densities at TSSs of genes with (red) and without (blue) identified daTSSs.
(TIF)

S5 Fig Plots of ChIP-seq read counts for histone modifications collected in HMEC cells. For each modification, observed gene TSS-centered heatmaps of ChIP-seq read counts are shown sorted by increasing distance to uaTSSs or daTSSs. Average densities are shown centered on uaTSS and daTSS positions (“uaTSS-centered” and “daTSS-centered”). To reflect the genomic context of transcription factor binding at promoters, plots of average density at antisense TSSs are transposed and shifted by median distance to gene TSSs (illustrated at bottom of figure). uaTSS- and daTSS-centered densities are plotted relative to observed gene TSS positions. In these plots (“Gene TSS-centered”), antisense plots were first transposed about the antisense TSS (left-most points became the right-most points and vice-versa) and then shifted by median distances observed between gene TSSs and antisense TSSs. Each plot considers 5,519 gene TSS-uaTSS or 2,956 gene TSS-daTSS pairs.
(TIF)

S6 Fig Plots of ChIP-seq read counts for transcription factors. TBP ChIP-seq data were collected in GM12878 cells; GATA3 in MCF7 cells; SP1 in A549 cells; NFIC in GM12878 cells; c-Fos in K562 cells; c-Jun in K562 cells (data sources outlined in S1 Table). For a detailed description of plots, see S5 Fig.
(TIF)

S7 Fig Plots of ChIP-seq read counts for chromatin remodelers. CHD1-A (HAT SAGA complex) ChIP-seq data were collected in K562 cells; Sap30 (Sin3-HDAC) in K562 cells; BRG1 (SWI/SNF) in HeLa cells; INI1 (SWI/SNF) in HeLa cells; BAF155 (SWI/SNF) in HeLa cells; BAF170 (SWI/SNF) in HeLa cells (data sources outlined in S1 Table). For a detailed description of plots, see S5 Fig.
(TIF)

S8 Fig Plots of ChIP-seq read counts for regulatory factors. p300 ChIP-seq data were collected in MCF7 cells; CTCF in A549 cells (data sources outlines in S1 Table). For a detailed description of plots, see S5 Fig.
(TIF)

S9 Fig Overview of genomic features observed at identified TSSs. For any given data type, as listed in the leftmost column, average read density or occurrences are plotted over a +/-1 kb window across TSS calls. Each plot is oriented relative to the gene with larger values corresponding to more downstream sequences relative to the gene TSS. Experimental cell lines are noted under the data type.
(TIF)

## References

[1] Butler JE, Kadonaga JT (2002) The RNA polymerase II core promoter: a key component in the regulation of gene expression. Genes Dev 16: 2583–2592. 12381658 10.1101/gad.1026202

[2] Boyle AP, Davis S, Shulha HP, Meltzer P, Margulies EH, et al (2008) High-resolution mapping and characterization of open chromatin across the genome. Cell 132: 311–322. 10.1016/j.cell.2007.12.014 18243105 PMC2669738

[3] Lenhard B, Sandelin A, Carninci P (2012) Metazoan promoters: emerging characteristics and insights into transcriptional regulation. Nat Rev Genet 13: 233–245. 10.1038/nrg3163 22392219

[4] Core LJ, Waterfall JJ, Lis JT (2008) Nascent RNA sequencing reveals widespread pausing and divergent initiation at human promoters. Science 322: 1845–1848. 10.1126/science.1162228 19056941 PMC2833333

[5] Preker P, Nielsen J, Kammler S, Lykke-Andersen S, Christensen MS, et al (2008) RNA exosome depletion reveals transcription upstream of active human promoters. Science 322: 1851–1854. 10.1126/science.1164096 19056938

[6] Seila AC, Calabrese JM, Levine SS, Yeo GW, Rahl PB, et al (2008) Divergent transcription from active promoters. Science 322: 1849–1851. 10.1126/science.1162253 19056940 PMC2692996

[7] Core LJ, Martins AL, Danko CG, Waters CT, Siepel A, et al (2014) Analysis of nascent RNA identifies a unified architecture of initiation regions at mammalian promoters and enhancers. Nat Genet 46: 1311–1320. 10.1038/ng.3142 25383968 PMC4254663

[8] Flynn RA, Almada AE, Zamudio JR, Sharp PA (2011) Antisense RNA polymerase II divergent transcripts are P-TEFb dependent and substrates for the RNA exosome. Proc Natl Acad Sci U S A 108: 10460–10465. 10.1073/pnas.1106630108 21670248 PMC3127934

[9] Ntini E, Jarvelin AI, Bornholdt J, Chen Y, Boyd M, et al (2013) Polyadenylation site-induced decay of upstream transcripts enforces promoter directionality. Nat Struct Mol Biol 20: 923–928. 10.1038/nsmb.2640 23851456

[10] Duttke SH, Lacadie SA, Ibrahim MM, Glass CK, Corcoran DL, et al (2015) Human promoters are intrinsically directional. Mol Cell 57: 674–684. 10.1016/j.molcel.2014.12.029 25639469 PMC4336624

[11] Scruggs BS, Gilchrist DA, Nechaev S, Muse GW, Burkholder A, et al (2015) Bidirectional Transcription Arises from Two Distinct Hubs of Transcription Factor Binding and Active Chromatin. Mol Cell 58: 1101–1112. 10.1016/j.molcel.2015.04.006 26028540 PMC4475495

[12] Minchiotti G, Di Nocera PP (1991) Convergent transcription initiates from oppositely oriented promoters within the 5' end regions of Drosophila melanogaster F elements. Mol Cell Biol 11: 5171–5180. 1656225 10.1128/mcb.11.10.5171-5180.1991 PMC361545

[13] Xu Z, Wei W, Gagneur J, Perocchi F, Clauder-Munster S, et al (2009) Bidirectional promoters generate pervasive transcription in yeast. Nature 457: 1033–1037. 10.1038/nature07728 19169243 PMC2766638

[14] Djebali S, Davis CA, Merkel A, Dobin A, Lassmann T, et al (2012) Landscape of transcription in human cells. Nature 489: 101–108. 10.1038/nature11233 22955620 PMC3684276

[15] Mayer A, di Iulio J, Maleri S, Eser U, Vierstra J, et al (2015) Native elongating transcript sequencing reveals human transcriptional activity at nucleotide resolution. Cell 161: 541–554. 10.1016/j.cell.2015.03.010 25910208 PMC4528962

[16] Nechaev S, Fargo DC, dos Santos G, Liu L, Gao Y, et al (2010) Global analysis of short RNAs reveals widespread promoter-proximal stalling and arrest of Pol II in Drosophila. Science 327: 335–338. 10.1126/science.1181421 20007866 PMC3435875

[17] Gardiner-Garden M, Frommer M (1987) CpG islands in vertebrate genomes. J Mol Biol 196: 261–282. 3656447 10.1016/0022-2836(87)90689-9

[18] Mathelier A, Zhao X, Zhang AW, Parcy F, Worsley-Hunt R, et al (2014) JASPAR 2014: an extensively expanded and updated open-access database of transcription factor binding profiles. Nucleic Acids Res 42: D142–147. 10.1093/nar/gkt997 24194598 PMC3965086

[19] Siepel A, Bejerano G, Pedersen JS, Hinrichs AS, Hou M, et al (2005) Evolutionarily conserved elements in vertebrate, insect, worm, and yeast genomes. Genome Res 15: 1034–1050. 16024819 10.1101/gr.3715005 PMC1182216

[20] Bucher P (1990) Weight matrix descriptions of four eukaryotic RNA polymerase II promoter elements derived from 502 unrelated promoter sequences. J Mol Biol 212: 563–578. 2329577 10.1016/0022-2836(90)90223-9

[21] Gilchrist DA, Adelman K (2012) Coupling polymerase pausing and chromatin landscapes for precise regulation of transcription. Biochim Biophys Acta 1819: 700–706. 10.1016/j.bbagrm.2012.02.015 22406341 PMC3371112

[22] Takaku M, Grimm SA, Shimbo T, Perera L, Menafra R, et al (2016) GATA3-dependent cellular reprogramming requires activation-domain dependent recruitment of a chromatin remodeler. Genome Biol 17: 36 10.1186/s13059-016-0897-0 26922637 PMC4769547

[23] Encode Project Consortium (2012) An integrated encyclopedia of DNA elements in the human genome. Nature 489: 57–74. 10.1038/nature11247 22955616 PMC3439153

[24] Burd CJ, Ward JM, Crusselle-Davis VJ, Kissling GE, Phadke D, et al (2012) Analysis of chromatin dynamics during glucocorticoid receptor activation. Mol Cell Biol 32: 1805–1817. 10.1128/MCB.06206-11 22451486 PMC3347412

[25] Goodman RH, Smolik S (2000) CBP/p300 in cell growth, transformation, and development. Genes Dev 14: 1553–1577. 10887150

[26] Kim T, Xu Z, Clauder-Munster S, Steinmetz LM, Buratowski S (2012) Set3 HDAC mediates effects of overlapping noncoding transcription on gene induction kinetics. Cell 150: 1158–1169. 10.1016/j.cell.2012.08.016 22959268 PMC3461055

[27] Shearwin KE, Callen BP, Egan JB (2005) Transcriptional interference—a crash course. Trends Genet 21: 339–345. 15922833 10.1016/j.tig.2005.04.009 PMC2941638

[28] Carissimi C, Laudadio I, Cipolletta E, Gioiosa S, Mihailovich M, et al (2015) ARGONAUTE2 cooperates with SWI/SNF complex to determine nucleosome occupancy at human Transcription Start Sites. Nucleic Acids Res 43: 1498–1512. 10.1093/nar/gku1387 25605800 PMC4330357

[29] Fryer CJ, Archer TK (1998) Chromatin remodelling by the glucocorticoid receptor requires the BRG1 complex. Nature 393: 88–91. 9590696 10.1038/30032

[30] Martin M (2011) Cutadapt removes adapter sequences from high-throughput sequencing reads. EMBnet 17: 10–12.

[31] Langmead B, Trapnell C, Pop M, Salzberg SL (2009) Ultrafast and memory-efficient alignment of short DNA sequences to the human genome. Genome Biol 10: R25 10.1186/gb-2009-10-3-r25 19261174 PMC2690996

[32] Pruitt KD, Brown GR, Hiatt SM, Thibaud-Nissen F, Astashyn A, et al (2014) RefSeq: an update on mammalian reference sequences. Nucleic Acids Res 42: D756–763. 10.1093/nar/gkt1114 24259432 PMC3965018

[33] Jothi R, Cuddapah S, Barski A, Cui K, Zhao K (2008) Genome-wide identification of in vivo protein-DNA binding sites from ChIP-Seq data. Nucleic Acids Res 36: 5221–5231. 10.1093/nar/gkn488 18684996 PMC2532738

[34] Heinz S, Benner C, Spann N, Bertolino E, Lin YC, et al (2010) Simple combinations of lineage-determining transcription factors prime cis-regulatory elements required for macrophage and B cell identities. Mol Cell 38: 576–589. 10.1016/j.molcel.2010.05.004 20513432 PMC2898526

[35] Crooks GE, Hon G, Chandonia JM, Brenner SE (2004) WebLogo: a sequence logo generator. Genome Res 14: 1188–1190. 15173120 10.1101/gr.849004 PMC419797

[36] Grant CE, Bailey TL, Noble WS (2011) FIMO: scanning for occurrences of a given motif. Bioinformatics 27: 1017–1018. 10.1093/bioinformatics/btr064 21330290 PMC3065696

[37] Bailey TL, Boden M, Buske FA, Frith M, Grant CE, et al (2009) MEME SUITE: tools for motif discovery and searching. Nucleic Acids Res 37: W202–208. 10.1093/nar/gkp335 19458158 PMC2703892

[38] QIAGEN (2016) Ingenuity Pathway Analysis. 27216297 version. Redwood City: QIAGEN.

[39] The Broad Institute (2015) Picard. 1.118 version Cambridge: The Broad Institute.

[40] Kim D, Pertea G, Trapnell C, Pimentel H, Kelley R, et al (2013) TopHat2: accurate alignment of transcriptomes in the presence of insertions, deletions and gene fusions. Genome Biol 14: R36 10.1186/gb-2013-14-4-r36 23618408 PMC4053844

[41] Trapnell C, Roberts A, Goff L, Pertea G, Kim D, et al (2012) Differential gene and transcript expression analysis of RNA-seq experiments with TopHat and Cufflinks. Nat Protoc 7: 562–578. 10.1038/nprot.2012.016 22383036 PMC3334321

[42] Ramirez F, Dundar F, Diehl S, Gruning BA, Manke T (2014) deepTools: a flexible platform for exploring deep-sequencing data. Nucleic Acids Res 42: W187–191. 10.1093/nar/gku365 24799436 PMC4086134

[43] Partek Inc. (2014) Partek Genomics Suite. 6.6 version St. Louis: Partek Inc.

