# Supplementary material for: Downstream Antisense Transcription Predicts Genomic Features That Define the Specific Chromatin Environment at Mammalian Promoters
Source: PLoS Genet. 2016 Aug 3;12(8):e1006224. doi: 10.1371/journal.pgen.1006224 (PMC4972320; doi:10.1371/journal.pgen.1006224)
Supplement: S1 Table — (PDF) [file pgen.1006224.s001.pdf]

**S1 Table. Sources of public data.**

| Experimental data type | Cell line | GEO/SRA ID(s) | Reference |
|---|---|---|---|
| Pol II ChIP-seq | MCF7 | GSM822295 | [1] |
| H2A.Z ChIP-seq | HMEC | GSM1003500 | [1] |
| H3K4me3 ChIP-seq | HMEC | GSM733712 | [1] |
| H3K27ac ChIP-seq | HMEC | GSM733660 | [1] |
| H3K4me1 ChIP-seq | HMEC | GSM733705 | [1] |
| HeK36me3 ChIP-seq | HMEC | GSM733707 | [1] |
| TBP ChIP-seq | GM12878 | GSM935277 | [1] |
| GATA3 ChIP-seq | MCF7 | GSM1010783 | [1] |
| SP1 ChIP-seq | A549 | GSM1010813 | [1] |
| NFIC ChIP-seq | GM12878 | GSM1010881 | [1] |
| c-Fos ChIP-seq | K562 | GSM935355 | [1] |
| c-Jun ChIP-seq | K562 | GSM935411 | [1] |
| p300 ChIP-seq | MCF7 | GSM1010800 | [1] |
| CHD1-A ChIP-seq | K562 | GSM1003575 | [1] |
| Sap30 ChIP-seq | K562 | GSM1003445 | [1] |
| BRG1 ChIP-seq | HeLa | GSM935511 | [1] |
| INI1 ChIP-seq | HeLa | GSM935634 | [1] |
| BAF155 ChIP-seq | HeLa | GSM935637 | [1] |
| BAF170 ChIP-seq | HeLa | GSM935638 | [1] |
| CTCF ChIP-seq | A549 | GSM1003606 | [1] |
| FAIRE-seq | T-47D/A1-2 | SRA044977 | [2] |
| MNase-seq | MCF7 | GSM1856003, GSM1856004, GSM1856005 | [3] |
| Pol II ChIP-seq | GM12878 | GSM935386 | [1] |
| Pol II ChIP-seq | K562 | GSM935358 | [1] |
| NET-seq | HeLa | GSM1505438, GSM1505439 | [4] |
| NET-seq | HEK239T | GSM1505440, GSM1505441 | [4] |
| GRO-seq | GM12878 | GSM1480326 | [5] |
| GRO-seq | K562 | GSM1480325 | [5] |
| GRO-cap | GM12878 | GSM1480323 | [5] |
| GRO-cap | K562 | GSM1480321 | [5] |
| PRO-seq | K562 | GSM1480327 | [5] |
| SPT5 ChIP-seq | HeLa | GSM1280295 | [6] |
| NELF-A ChIP-seq | HeLa | GSM1280296 | [6] |
| NELF-E ChIP-seq | K562 | GSM935392 | [1] |
| Ccnt2 ChIP-seq | K562 | GSM935547 | [1] |


1. Encode Project Consortium (2012) An integrated encyclopedia of DNA elements in the human genome. Nature 489: 57-74.
2. Burd CJ, Ward JM, Crusselle-Davis VJ, Kissling GE, Phadke D, et al. (2012) Analysis of chromatin dynamics during glucocorticoid receptor activation. Mol Cell Biol 32: 1805-1817.
3. Takaku M, Grimm SA, Shimbo T, Perera L, Menafra R, et al. (2016) GATA3-dependent cellular reprogramming requires activation-domain dependent recruitment of a chromatin remodeler. Genome Biol 17: 36.
4. Mayer A, di Iulio J, Maleri S, Eser U, Vierstra J, et al. (2015) Native elongating transcript sequencing reveals human transcriptional activity at nucleotide resolution. Cell 161: 541-554.
5. Core LJ, Martins AL, Danko CG, Waters CT, Siepel A, et al. (2014) Analysis of nascent RNA identifies a unified architecture of initiation regions at mammalian promoters and enhancers. Nat Genet 46: 1311-1320.

6. Liu P, Xiang Y, Fujinaga K, Bartholomeeusen K, Nilson KA, et al. (2014) Release of positive transcription elongation factor b (P-TEFb) from 7SK small nuclear ribonucleoprotein (snRNP) activates hexamethylene bisacetamide-inducible protein (HEXIM1) transcription. J Biol Chem 289: 9918-9925.
