# Supplementary material for: Downstream Antisense Transcription Predicts Genomic Features That Define the Specific Chromatin Environment at Mammalian Promoters
Source: PLoS Genet. 2016 Aug 3;12(8):e1006224. doi: 10.1371/journal.pgen.1006224 (PMC4972320; doi:10.1371/journal.pgen.1006224)
Supplement: S2 Table — (PDF) [file pgen.1006224.s002.pdf]

S2 Table. Enrichment test p-values for ChIP-seq data sets.

ChIP-seq experiment	p-value (Two-tailed Wilcoxon)	Reference
SPT5	2.65×10^{-33}	[1]
NELF-A	1.25×10^{-37}	[1]
NELF-E	1.17×10^{-32}	[2]
Ccnt2	2.27×10^{-63}	[2]
H2A.Z	6.60×10^{-10}	[2]
H3K27ac	1.17×10^{-3}	[2]
H3K4me3	3.95×10^{-2}	[2]
H3K9ac	0.978	[2]
H3K4me1	0.530	[2]
HeK36me3	0.442	[2]
TBP	6.04×10^{-27}	[2]
GATA3	2.07×10^{-116}	[2]
SP1	4.91×10^{-47}	[2]
NFIC	1.67×10^{-17}	[2]
c-Fos	1.22×10^{-18}	[2]
c-Jun	3.57×10^{-52}	[2]
p300	8.32×10^{-81}	[2]
CHD1-A	6.51×10^{-09}	[2]
Sap30	7.58×10^{-16}	[2]
BRG1	5.43×10^{-14}	[2]
INI1	3.32×10^{-68}	[2]
BAF155	7.60×10^{-28}	[2]
BAF170	1.98×10^{-18}	[2]
CTCF	2.85×10^{-26}	[2]

1. Liu P, Xiang Y, Fujinaga K, Bartholomeeusen K, Nilson KA, et al. (2014) Release of positive transcription elongation factor b (P-TEFb) from 7SK small nuclear ribonucleoprotein (snRNP) activates hexamethylene bisacetamide-inducible protein (HEXIM1) transcription. J Biol Chem 289: 9918-9925.
2. Encode Project Consortium (2012) An integrated encyclopedia of DNA elements in the human genome. Nature 489: 57-74.
