# Supplementary material for: Downstream Antisense Transcription Predicts Genomic Features That Define the Specific Chromatin Environment at Mammalian Promoters
Source: PLoS Genet. 2016 Aug 3;12(8):e1006224. doi: 10.1371/journal.pgen.1006224 (PMC4972320; doi:10.1371/journal.pgen.1006224)
Supplement: S3 Table — (PDF) [file pgen.1006224.s003.pdf]

S3 Table. Overlap of daTSSs with RefSeq-annotated exons and introns. A daTSS was considered overlapping an exon or intron if the daTSS was positioned within any exon or intron in the reference annotation. To test relative enrichment, daTSSs were shuffled amongst gene models, maintaining the same distribution of gene TSS-daTSS distances observed in the experimental data set. We report the average and standard deviation of overlapped daTSSs over 200 random shuffles.

Identified daTSS positions		Shuffled daTSS positions (200x)	
Exon overlap	Intron overlap	Exon overlap	Intron overlap
720 (24%)	2,202 (74%)	712.3 ± 16.8	2,221.2 ± 14.6
