# Supplementary figures and images for: Downstream Antisense Transcription Predicts Genomic Features That Define the Specific Chromatin Environment at Mammalian Promoters
Source: PLoS Genet. 2016 Aug 3;12(8):e1006224. doi: 10.1371/journal.pgen.1006224 (PMC4972320; doi:10.1371/journal.pgen.1006224)

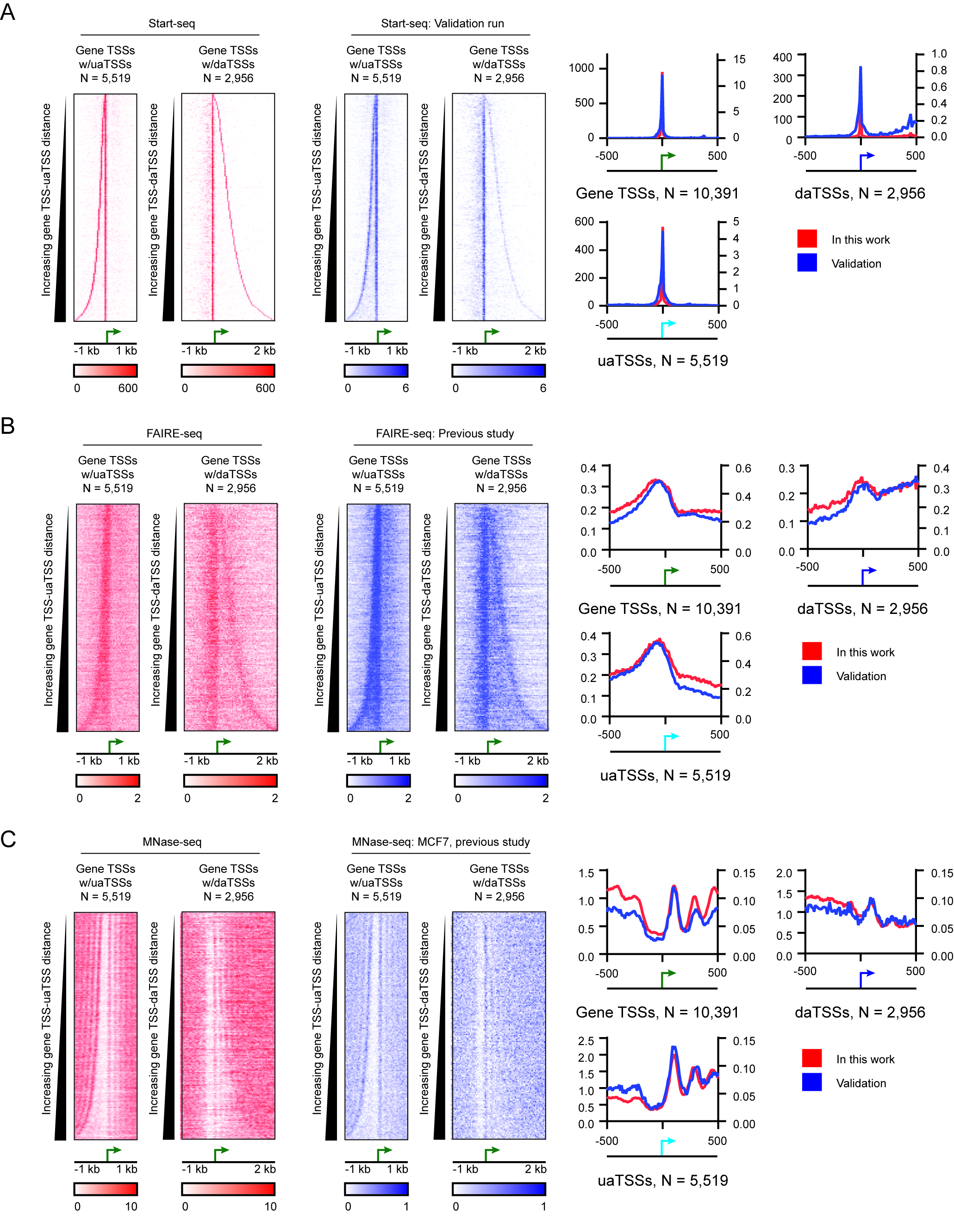

Supplement: S1 Fig — Reported data (red) and validation data (blue) are shown anchored at identified TSS positions in both heatmaps and two-dimensional plots. Validation data are taken from biological replicates or from similar cell lines. Read density of a Start-seq biological replicate (T-47D/A1-2 cells) (A), FAIRE-seq in T-47D/A1-2 cells [24] (B), and of MNase-seq in MCF-7 cells [22] (C) are shown at identified TSS positions. (TIF) [file pgen.1006224.s004.tif]

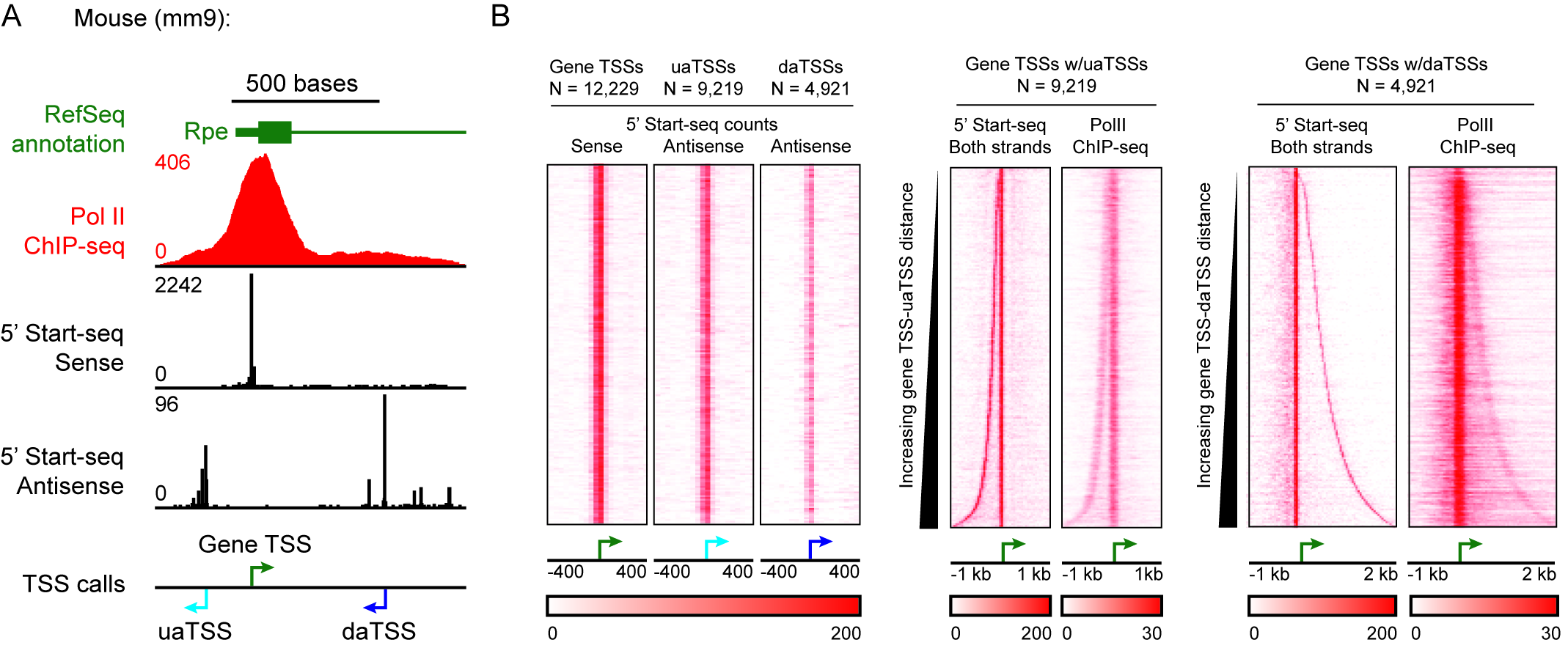

Supplement: S2 Fig — (A) Observed transcription at gene Rpe. Genomic context is given by RefSeq annotation (green), Pol II ChIP-seq coverage (red), and counts for the 5’ ends of Start-seq reads in both sense and anti-sense orientations (black). Observed transcription start sites (TSSs) are given for the gene TSS, upstream antisense TSS (uaTSS), and downstream antisense TSS (daTSS). (B) Heatmaps of counts for the 5’ ends of Start-seq reads over gene TSS, uaTSS, and daTSS positions. (Right panel) Heatmaps of counts for the 5’ ends of Start-seq reads and fragment centers of Pol II ChIP-seq reads. Heatmaps are centered on gene TSS positions and sorted by gene TSS-uaTSS (left) or gene TSS-daTSS distance (right). Only TSS positions with called uaTSS or daTSS positions, respectively, are included on the heatmaps. (TIF) [file pgen.1006224.s005.tif]

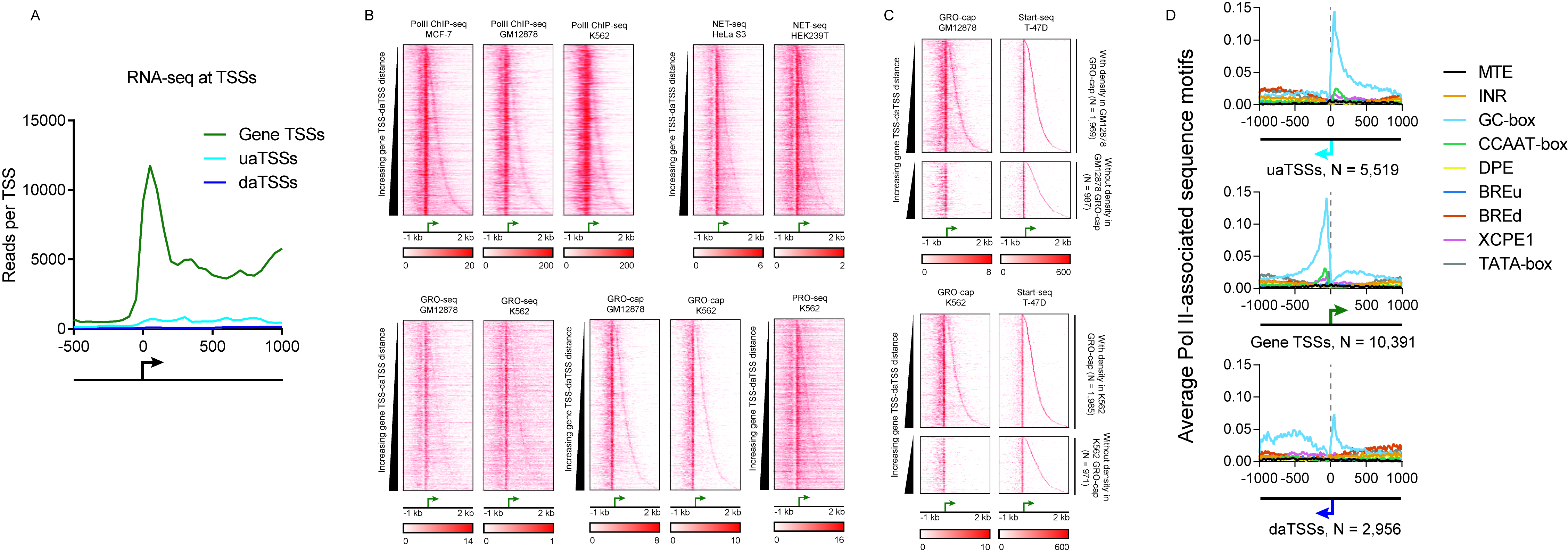

Supplement: S3 Fig — (A) Average RNA-seq coverage in T47D/A1-2 cells across identified TSS positions. (B) Heatmaps of read density from Pol II-associated sequencing approaches. Pol II ChIP-seq, NET-seq, GRO-seq, GRO-cap, and PRO-seq were performed over a variety of cell lines (indicated on figure). Each heatmap is centered on observed gene TSS position and sorted by increasing gene TSS-daTSS distance. (C) Categorization of T47D/A1-2-called daTSSs by presence of GRO-cap signal in heterologous cell lines. daTSSs were placed into a separate category if no GRO-cap signal was found within 10 nt of the observed daTSS. 987 (33%) and 971 (33%) daTSSs called in T47D/A1-2 cells were found to have no significant GRO-cap signal in GM12878 and K562 samples, respectively. (D) Plots of average occurrences of Pol II-associated sequence motifs. Motif occurrences were identified using FIMO [36]. Motif position weight matrices were taken from the Pol II subset of the JASPAR database [18]. (TIF) [file pgen.1006224.s006.tif]

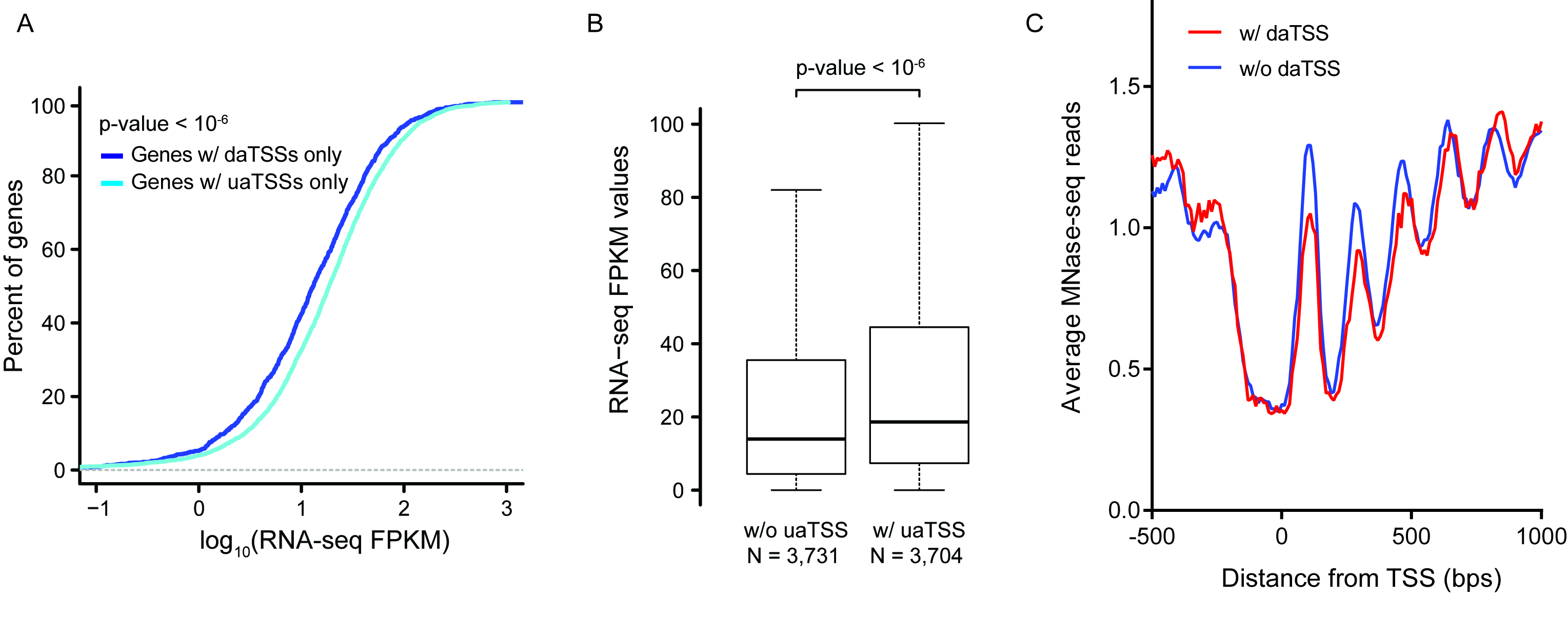

Supplement: S4 Fig — (A) Empirical cumulative distributions of gene RNA-seq FPKM values for genes displaying only daTSSs (blue) and only uaTSSs (cyan). Inset p-value was determined by Kolmogorov-Smirnov test. (B) Box plots of RNA-seq FPKM values for all genes, genes without uaTSSs, and genes without daTSSs. Reported p-values were determined by Kolmgorov-Smirnov tests. (C) Average MNase-seq read densities at TSSs of genes with (red) and without (blue) identified daTSSs. (TIF) [file pgen.1006224.s007.tif]

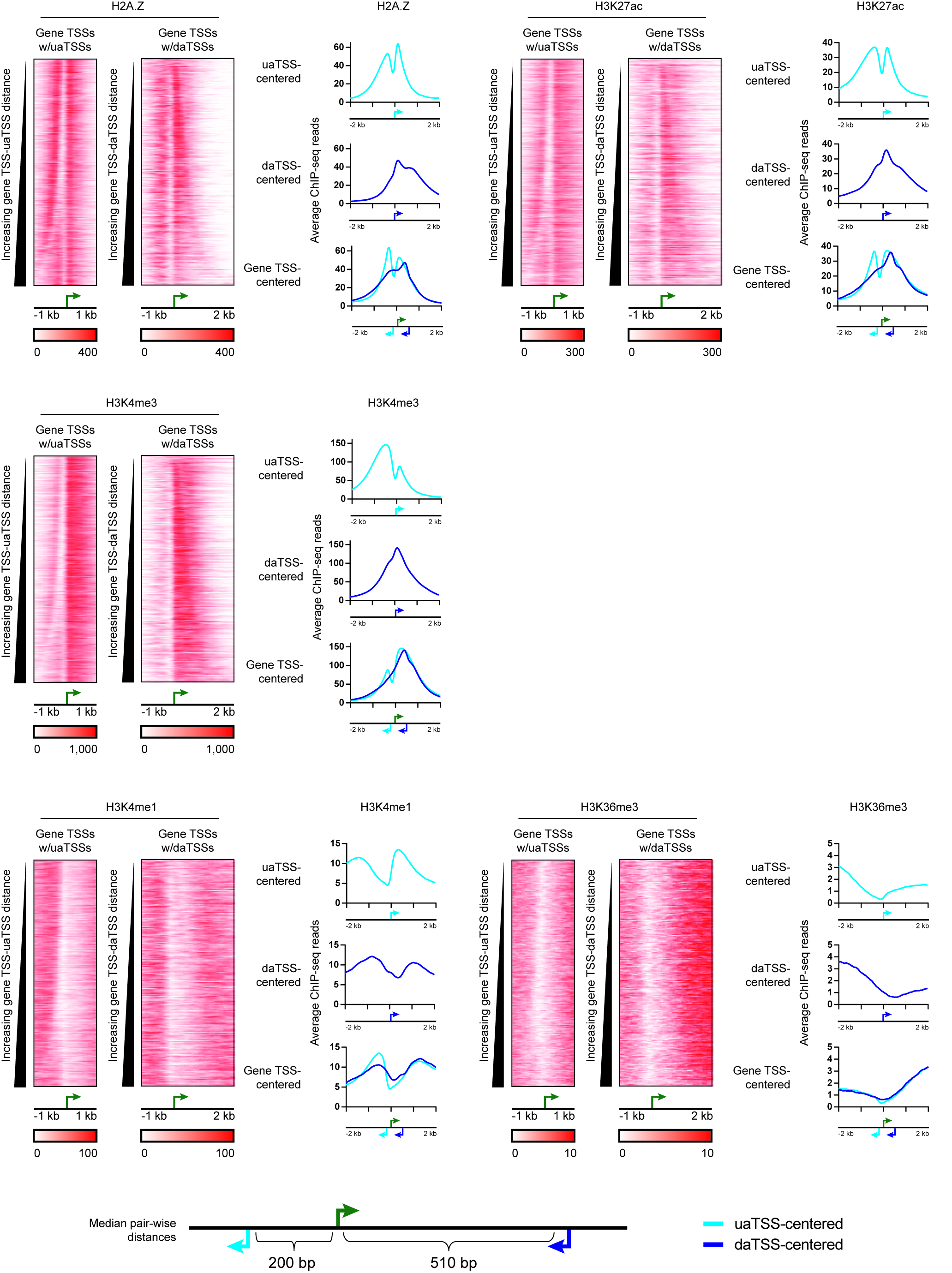

Supplement: S5 Fig — For each modification, observed gene TSS-centered heatmaps of ChIP-seq read counts are shown sorted by increasing distance to uaTSSs or daTSSs. Average densities are shown centered on uaTSS and daTSS positions (“uaTSS-centered” and “daTSS-centered”). To reflect the genomic context of transcription factor binding at promoters, plots of average density at antisense TSSs are transposed and shifted by median distance to gene TSSs (illustrated at bottom of figure). uaTSS- and daTSS-centered densities are plotted relative to observed gene TSS positions. In these plots (“Gene TSS-centered”), antisense plots were first transposed about the antisense TSS (left-most points became the right-most points and vice-versa) and then shifted by median distances observed between gene TSSs and antisense TSSs. Each plot considers 5,519 gene TSS-uaTSS or 2,956 gene TSS-daTSS pairs. (TIF) [file pgen.1006224.s008.tif]

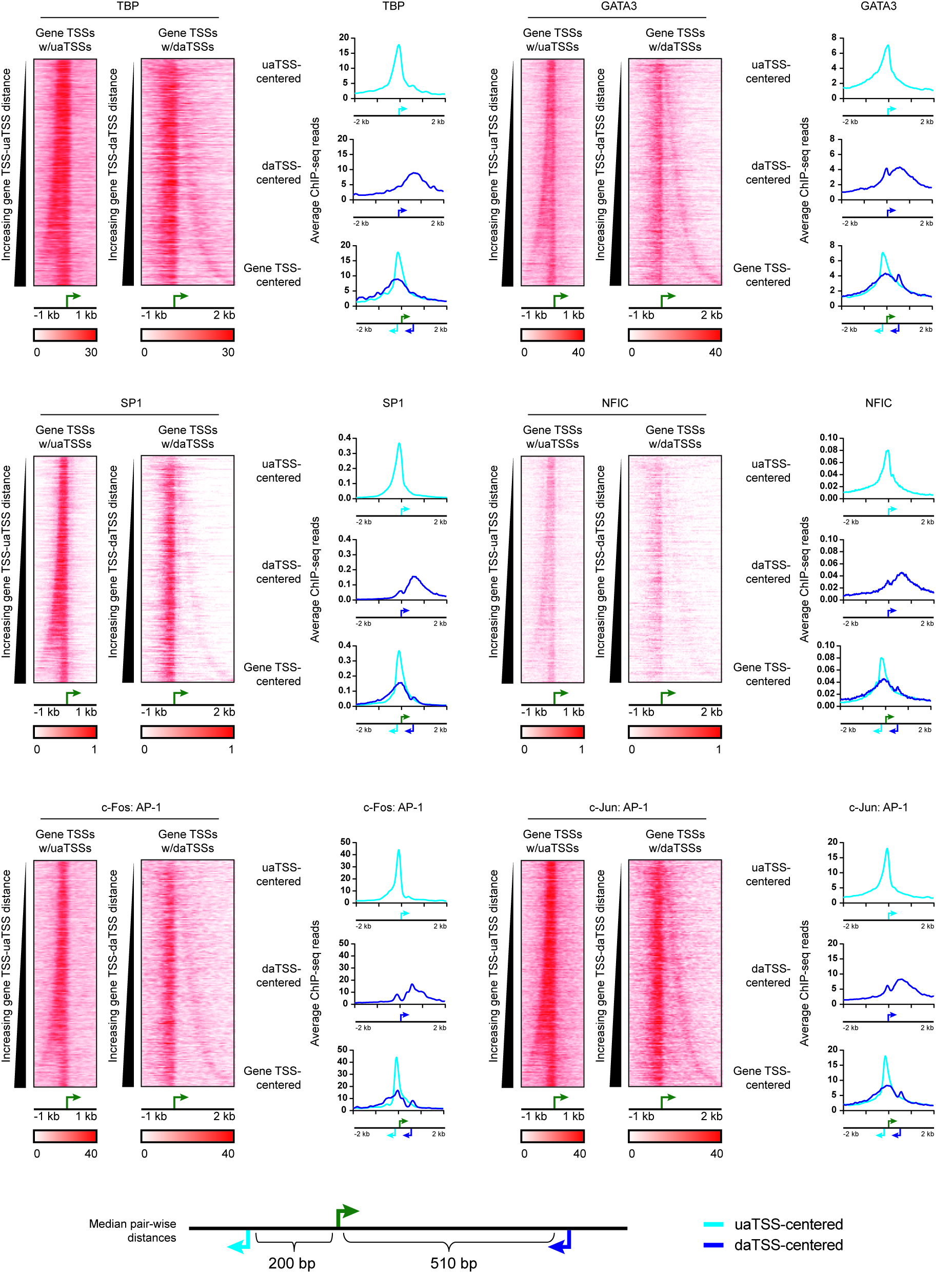

Supplement: S6 Fig — TBP ChIP-seq data were collected in GM12878 cells; GATA3 in MCF7 cells; SP1 in A549 cells; NFIC in GM12878 cells; c-Fos in K562 cells; c-Jun in K562 cells (data sources outlined in S1 Table). For a detailed description of plots, see S5 Fig. (TIF) [file pgen.1006224.s009.tif]

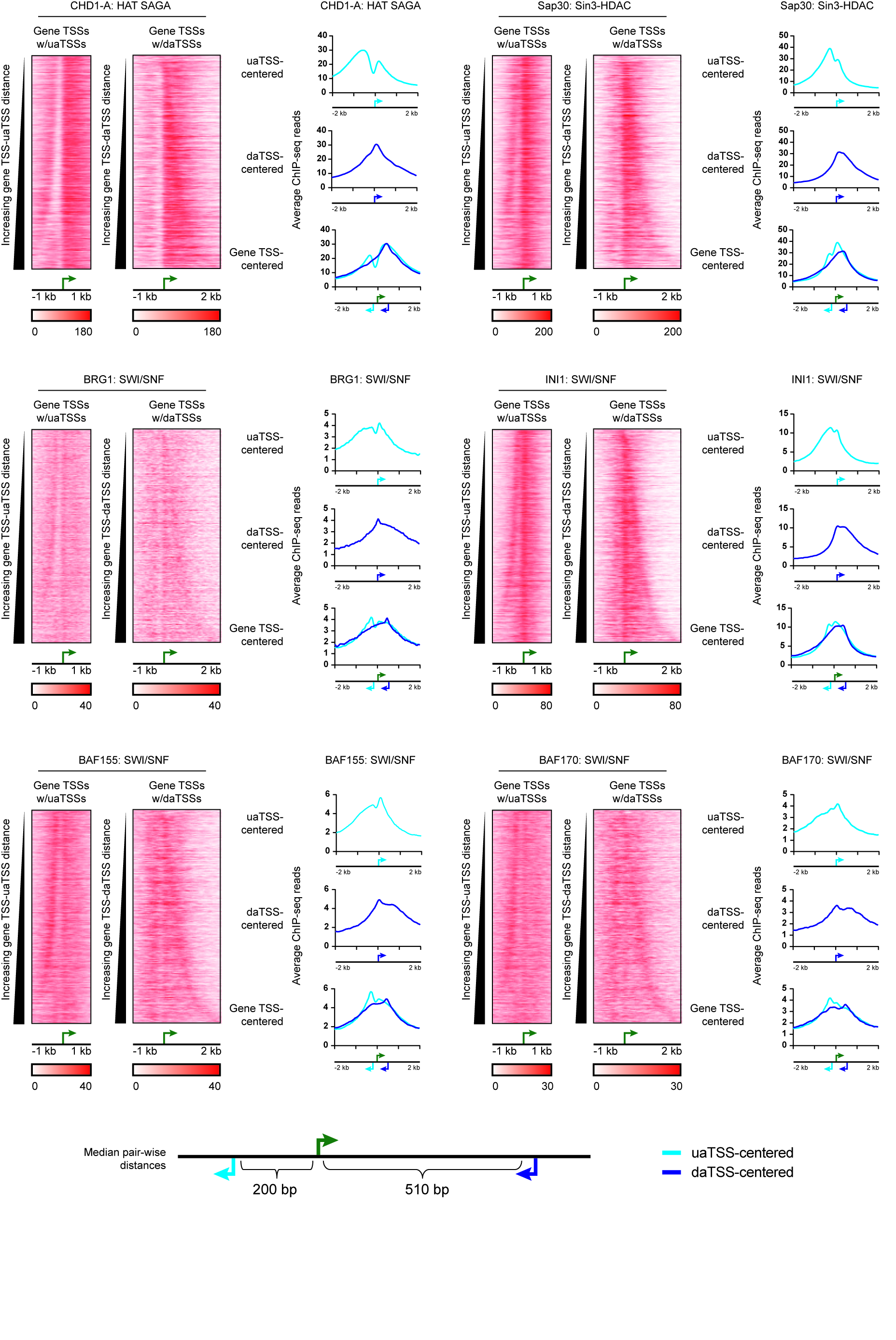

Supplement: S7 Fig — CHD1-A (HAT SAGA complex) ChIP-seq data were collected in K562 cells; Sap30 (Sin3-HDAC) in K562 cells; BRG1 (SWI/SNF) in HeLa cells; INI1 (SWI/SNF) in HeLa cells; BAF155 (SWI/SNF) in HeLa cells; BAF170 (SWI/SNF) in HeLa cells (data sources outlined in S1 Table). For a detailed description of plots, see S5 Fig. (TIF) [file pgen.1006224.s010.tif]

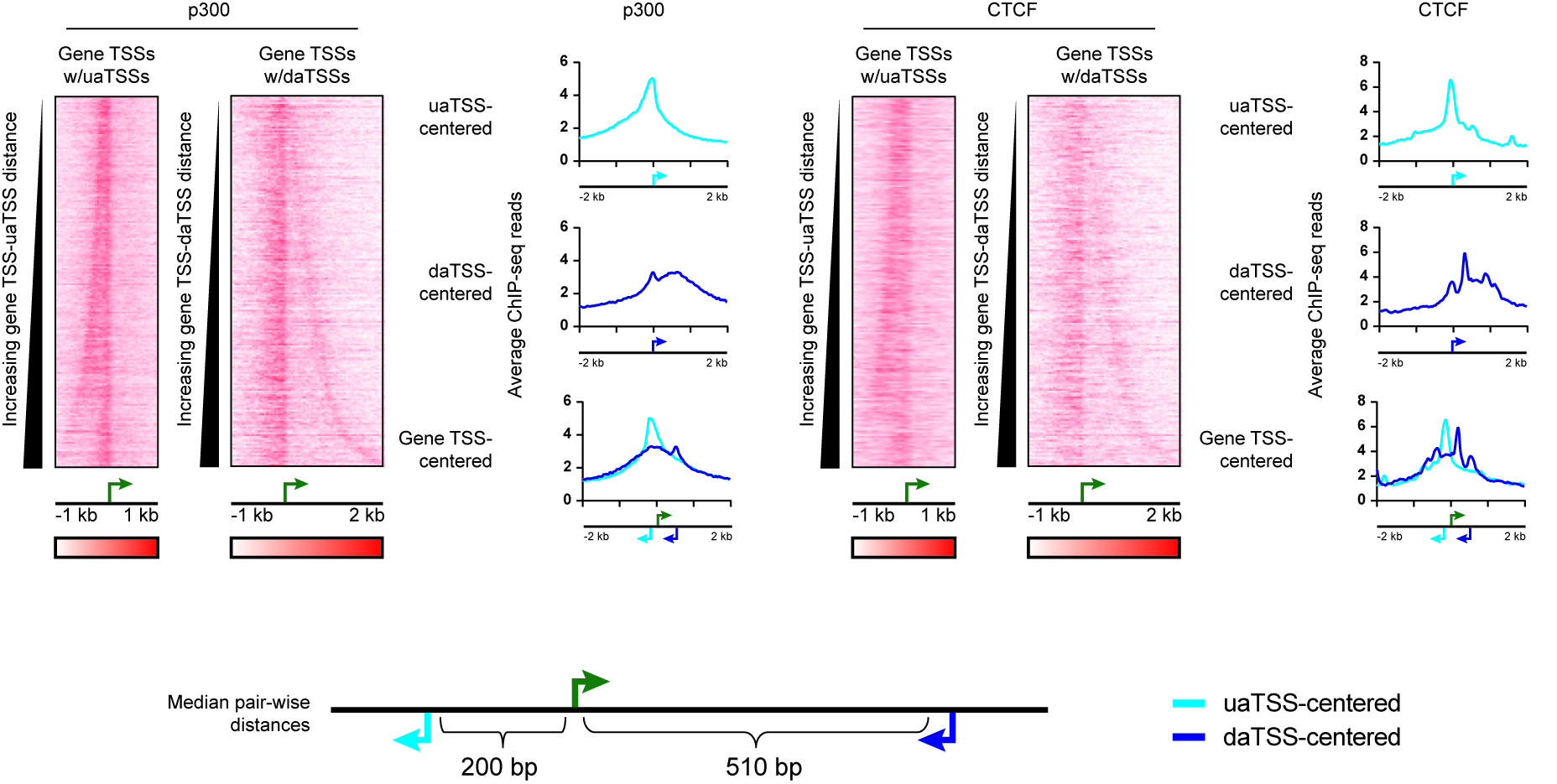

Supplement: S8 Fig — p300 ChIP-seq data were collected in MCF7 cells; CTCF in A549 cells (data sources outlines in S1 Table). For a detailed description of plots, see S5 Fig. (TIF) [file pgen.1006224.s011.tif]

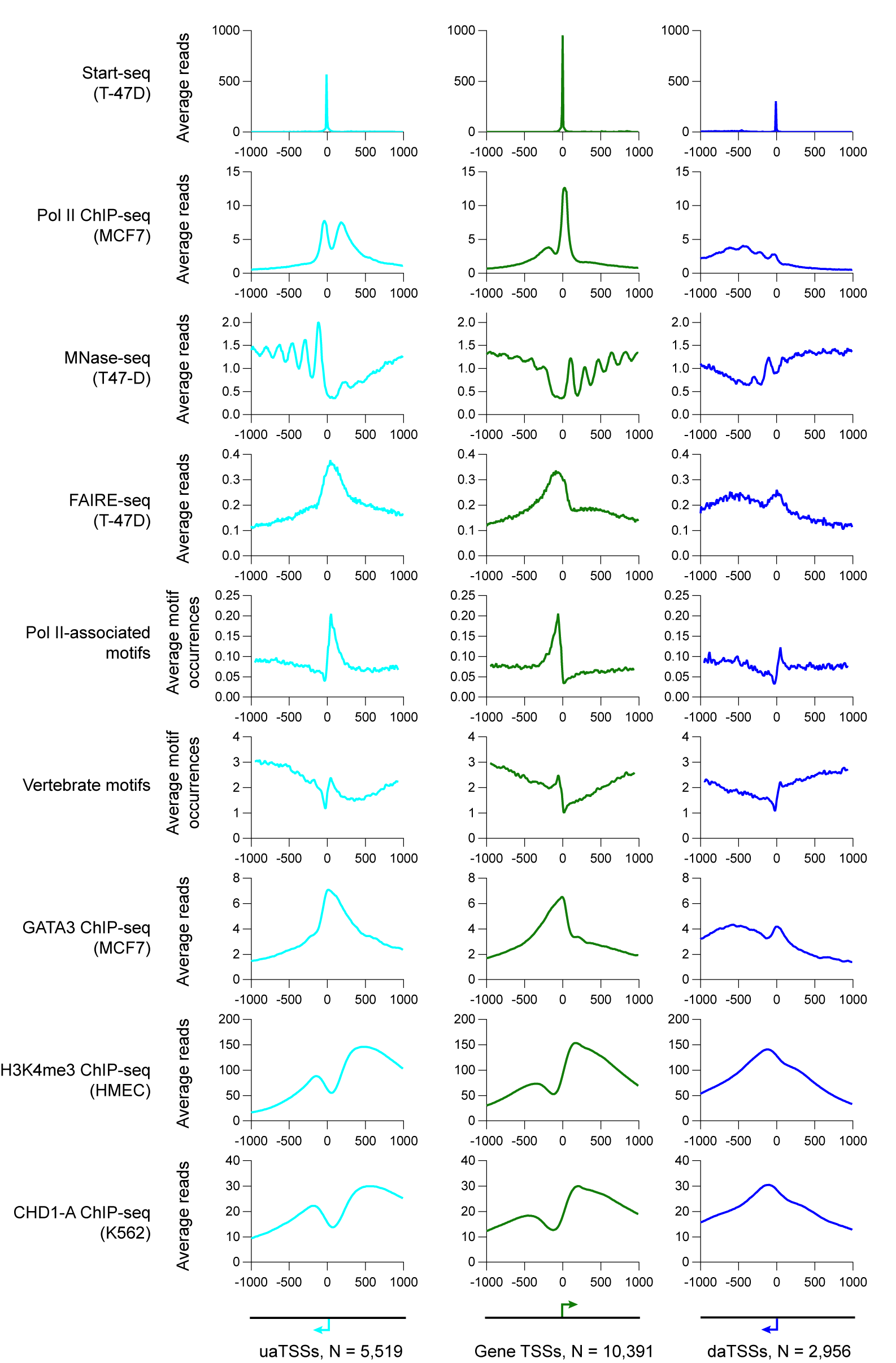

Supplement: S9 Fig — For any given data type, as listed in the leftmost column, average read density or occurrences are plotted over a +/-1 kb window across TSS calls. Each plot is oriented relative to the gene with larger values corresponding to more downstream sequences relative to the gene TSS. Experimental cell lines are noted under the data type. (TIF) [file pgen.1006224.s012.tif]
